# Metabolomics reveals that CAF-derived lipids promote colorectal cancer peritoneal metastasis by enhancing membrane fluidity

**DOI:** 10.7150/ijbs.68484

**Published:** 2022-02-21

**Authors:** Shaoyong Peng, Yingjie Li, Meijin Huang, Guannan Tang, Yumo Xie, Daici Chen, Yumin Hu, Tiantian Yu, Jian Cai, Zixu Yuan, Huaiming Wang, Hui Wang, Yanxin Luo, Xiaoxia Liu

**Affiliations:** 1 Department of Colon and Rectum Surgery, The Sixth Affiliated Hospital (Guangdong Gastrointestinal and Anal Hospital), Sun Yat-sen University, 26 Yuancun Erheng Road, Guangzhou, Guangdong, 510655, China.; 2 Guangdong Provincial Key Laboratory of Colorectal and Pelvic Floor Disease, The Sixth Affiliated Hospital (Guangdong Gastrointestinal and Anal Hospital), Sun Yat-sen University, Guangzhou, Guangdong, 510655, China.; 3 Department of Clinical Laboratory, The Sixth Affiliated Hospital, Sun Yat-sen University, Guangzhou, Guangdong, 510655, China.; 4 Sun Yat-sen University Metabolomics Center, Guangzhou, Guangdong, 510080, China.

**Keywords:** Lipidomics, colorectal cancer peritoneal metastasis, cancer-associated fibroblasts (CAFs), glucose metabolism, C16:0, metabolomics

## Abstract

Patients with peritoneal metastasis (PM) of colorectal cancer (CRC) have poorer overall survival outcomes than those without PM. Cancer-associated fibroblasts (CAFs) are a major component of the tumor microenvironment and mediate CRC progression and PM. It is imperative to identify and develop novel therapeutic targets for PM-CRC driven by CAFs. Using lipidomics, we reveal that the abundance of phosphatidylcholine (PC) with unsaturated acyl chains was increased in clinical PM-CRC specimens. Additionally, we found that CAFs were present at a higher relative abundance in primary PM-CRC tumors and that membrane fluidity in CRC cells was increased after incubation with CAF-conditioned medium (CM) through three independent methods: lipidomics, fluorescence recovery after photobleaching (FRAP), and generalized polarization. Then, we found that increased membrane fluidity can enhance glucose uptake and metabolism, as supported by real-time bioenergetics analysis and U-^13^C glucose labeling. Interestingly, stearoyl-CoA desaturase 1 (SCD), the rate-limiting enzyme in the biosynthesis of unsaturated fatty acids (uS-FAs), was expressed at low levels in PM and associated with poor prognosis in CRC patients. Importantly, by untargeted metabolomics analysis and fatty acid ([U-^13^C]-stearic acid) tracing analyses, we found that CRC cells take up lipids and lipid-like metabolites secreted from CAFs, which may compensate for low SCD expression. Both *in vitro* and *in vivo* experiments demonstrated that sodium palmitate (C16:0) treatment could decrease the CAF-induced change in cell membrane fluidity, limit glucose metabolism, suppress cell invasiveness, and impair tumor growth and intraperitoneal dissemination. An increased C16:0 concentration was shown to induce apoptosis linked to lipotoxicity. Furthermore, C16:0 effectively enhanced the antitumor activity of 5-fluorouracil (5-FU) *in vitro* and was well tolerated *in vivo*. Taken together, these findings suggest that adding the saturated fatty acid (S-FA) C16:0 to neoadjuvant chemotherapy may open new opportunities for treating PM-CRC in the future.

## Introduction

Colorectal cancer (CRC) is the third most commonly diagnosed malignancy and the second global cause of cancer death, but therapeutic options are still limited 1, 2. Metastasis to the peritoneum, the third most common site of colon cancer metastasis following the liver and lungs, has generally been associated with poorer prognosis and quality of life in the terminal stage of this disease 3-5. Although patients with peritoneal metastasis (PM) of colorectal cancer (PM-CRC) treated with cytoreductive surgery (CRS) and hyperthermic intraoperative intraperitoneal chemotherapy (HIPEC) show survival benefits, they continue to have poorer survival outcomes than those of patients without PM 4, 6, 7.

The tumor microenvironment has gained increasing attention in recent years, as it can potently influence the progression, metastasis, invasion, recurrence and therapeutic resistance of tumors 8, 9. As the most prevalent cell type and a vastly heterogeneous stromal cell population in the tumor microenvironment of solid tumors, cancer-associated fibroblasts (CAFs) were shown to enhance tumor growth, migration, invasion and immunosuppression by secreting cytokines, growth factors and chemokines 10-13. However, a growing number of studies have demonstrated that some CAFs function to suppress tumor growth 14, 15. Our previous study showed that enhanced CAF fatty acid (FA) catabolism can drive PM-CRC 16. The membrane lipid composition is critical to maintaining membrane fluidity, and decreasing membrane fluidity has been suggested as a new treatment approach for aggressive cancers 17. This study aimed to use a lipidomics approach to discover and validate novel therapeutic targets for PM-CRC. Here, we provide an understanding of the mechanisms responsible for the CAF-mediated regulation of CRC cell invasion and PM and explore strategies to enhance the treatment of patients with PM-CRC and improve patient survival.

## Materials and methods

### Clinical samples, isolation of CAFs and cell lines

All tumors came from patients with chemo-naïve, high-grade colorectal cancer (T4Nx) with an age range of 18 to 70 years. Primary tumors from CRC patients with and without PM were obtained from the Sixth Affiliated Hospital of Sun Yat-sen University (SYSU, China); a tissue microarray (TMA) was constructed from primary CRC tissues from patients that were resected at the Sixth Affiliated Hospital of Sun Yat-sen University. The Human Medical Ethics Committee of SYSU approved this study, and informed consent was obtained from all patients. CAFs were isolated from primary tumor samples, and control/conditioned media (CM) were collected as previously described 16. Briefly, CAFs were seeded and reached 90% confluence, and the medium was then replaced with complete DMEM supplemented with 1% pen/strep for 24 h. Media were collected and filtered with a 0.8-mm filter to obtain CM. Control CM was generated under the same conditions in a dish without cells.

The CRC cell lines HCT116 (RRID: CVCL_0291) and DLD1 (RRID: CVCL_0248) were obtained from the American Type Culture Collection (ATCC, USA) and cultured in McCoy's 5A and RPMI-1640 media (Gibco, Life Technologies, USA), respectively, with 10% fetal bovine serum (FBS) (Gibco, Life Technologies, USA). The cell lines were cultured in an incubator at 37 °C with 5% CO2. All cell lines were regularly tested for mycoplasma (every 3-4 months). All cell lines had been authenticated using short tandem repeat profiling within the last three years. All experiments were performed with mycoplasma-free cells.

Sodium palmitate (C16:0; Sigma-Aldrich (St. Louis, MO, USA)) was dissolved in methanol to a concentration of 40 mM by heating (52 °C) for up to 15 min to obtain a clear, colorless solution.

### Immunohistochemistry (IHC)

The α-smooth muscle actin (αSMA) and stearoyl-CoA desaturase 1 (SCD) proteins were stained according with a routine IHC method as previously described 16. Anti-αSMA antibodies were purchased from Cell Signaling Technologies (Danvers, MA, USA), and anti-SCD antibodies were purchased from Affinity Biosciences (OH, USA).

### Real-time bioenergetics analysis

Extracellular acidification rates (ECARs) were measured using an XF24 extracellular analyzer (Seahorse Bioscience, USA), as described previously 18.

### Glucose uptake and lactate production

Cells in the exponential growth phase were seeded in triplicate at a density of 2.5 × 10^4^ cells/mL in 24-well plates. Following 24 h of incubation at 37°C, CAF-CM (0.25 ml, 25%) and complete DMEM (0.75 ml, 75%) were added to each well and incubated for 48 h. Then, the medium was replaced with complete DMEM supplemented with 1% pen/strep, and the culture medium was collected after a 24-h incubation step to analyze glucose and lactate levels using a YSI 2950 STAT Plus instrument. Glucose uptake and lactate production were determined by calculating the differences in glucose and lactate concentrations between the cell culture medium and fresh medium without cells.

### Fluorescence recovery after photobleaching (FRAP)

After the indicated cells were attached to confocal dishes (NEST Biotechnology Co. Ltd., Wuxi, China), the lipid dye DiI (5 µg/mL) (Beyotime, China) was added, and the cells were cultured for another 1 h. Then, the DiI was washed off with warm PBS. FRAP images were acquired with an LSM880 confocal microscope equipped with a live-cell chamber (set at 37 °C and 5% CO_2_) and ZEN software (Zeiss) with a 40X water-immersion objective. Briefly, an area in the cell membrane for bleaching was selected. Then, the laser generated from a confocal scanning laser microscope at a power of 100% was used to bleach the area. After that, images of the bleached area at different time points were recorded. Images were captured, and the bleaching process was monitored. The fluorescence intensity of the bleached area was analyzed.

### Membrane fluidity of CRC cells

The membrane fluidity of DLD1 and HCT116 cells was measured by using the cell probe TMA-DPH (Sigma-Aldrich, St. Louis, MO, USA)^19^. Fluorescence measurements were performed on an RF-6000 spectrofluorophotometer with a polarizer (Shimadzu, Japan). CRC cells were treated with CAF-CM (25%) for 48 h; control medium was used as the control. After that, the cells were collected and washed with PBS. Then, the cells (4 × 10^6^) were labeled with TMA-DPH (1 μM) and incubated for 20 min at 37 °C to complete the labeling. Finally, the liquid was aspirated, and 3 mL of PBS was added to the cell suspensions. The degree of polarization (Pcorr) of TMA-DPH, which was used to detect the change in lipid fluidity, was measured with a fluorimeter and calculated using the following equation:



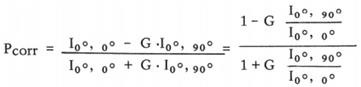



where Pcorr is the compensation degree of polarization and I_0_^0^ and I_90_^0^ are the fluorescence intensities measured by setting the excitation polarizer to the vertical orientation and the emission polarizer to the vertical and horizontal orientations, respectively. The G value was calculated as I_0_^0^/I_90_^0^.

### Apoptosis assay

Apoptosis was detected using an Annexin-V-FITC detection kit (BD Pharmingen™; San Diego, CA, USA) according to the manufacturer's instructions. The results were analyzed using CytExpert software.

### U-^13^C glucose labeling

CRC cells were treated with CAF-CM for 48 h and then cultured in glucose-free DMEM (Thermo Fisher Scientific) supplemented with 4.5 g/L U-^13^C-glucose (Cambridge Isotope Laboratories, Andover, MA) and 10% FBS for 24 h. Then, the medium was removed, and the cells in the culture dish were washed twice with 2 ml of saline without disturbing cell attachment. Next, 500 µL of methanol was added to the cells to quench the metabolic reactions, and an equal volume of water was added. The cells were then collected by scraping and placed in 2-ml Eppendorf tubes, and 500 µL of chloroform was added to each tube. The cell extracts were vortexed at 4°C for 30 min. The samples were centrifuged at 14,000 ×g for 5 min at room temperature. To analyze polar metabolites, the upper layer of the aqueous phase (700 µl) was first transferred to a new tube for evaporation under airflow (N2 gas or vacuum concentrator: 3 h, 45 °C). The dried metabolites were stored at -80 °C until LC/MS analysis. LC/MS was performed at the Metabolic Innovation Center of Sun Yat-Sen University.

### Cell migration and invasion assays

Transwell assays were performed with 24-well cell culture inserts with an 8.0-µm pore size (Falcon, USA) with Matrigel (1:10) (BD Biosciences, USA) as previously described 16. Each assay was repeated three times.

### Cell proliferation assay: CCK-8 and IncuCyte ZOOM

Cell proliferation was assessed by the Cell Counting Kit-8 (CCK-8) (Dojindo, Japan) and IncuCyte ZOOM as previously described 16.

### 2-NBDG uptake

A total of 2 × 10^5^ cells were incubated in DMEM (no glucose, 1% BSA) containing 1 mM 2-NBDG (Thermo Fisher Scientific) for 30 min at 37 °C in a cell incubator to measure glucose uptake. The cells were analyzed by fluorescence microscopy (Olympus, Tokyo, Japan).

### Knockdown assays

Lentiviral particles containing shRNAs against SCD and control shRNA were obtained from GeneCopoeia; the shRNAs had the following sequences: shSCD-1: CCTTTATGATGCTAAGCTGAT, shSCD-2: CCTATGACCGGAAGAAAGTCT, and shCTR: GCTTCGCGCCGTAGTCTTA.

### Label-free proteomic analysis

DLD1 cells in the exponential growth phase were seeded in triplicate at a density of 1.5 × 10^5^ cells/mL in 6-well plates. Following 24 h of incubation at 37°C, CAF-CM (0.5 ml, 25%) or control CM (0.5 ml, 25%) and complete DMEM (1.5 ml, 75%) were added to each well for 48 h. Then, the cells were collected. The proteomic experiment was performed as previously described 20, and data analysis was supported by Wayen Biotechnologies Co., Ltd. (Shanghai, China).

### Lipidomics

The lipid analysis of clinical tumor samples was performed by LC-MS/MS as described in a previous publication 16. For the lipidomics analysis of HCT116 cells, briefly, CAF-CM (25%, 2.5 ml) or CM (25%, 2.5 ml) and complete DMEM (7.5 ml, 75%) were added to HCT116 cells at approximately 30% confluence in a 10-cm dish for 48 h. Then, cells (5×10^6^) were collected (5 min at 14 000 ×g, 4 °C), washed with cold PBS, and snap-frozen in liquid nitrogen. Quintuplicate samples were sent for lipidomics analysis (Applied Protein Technology, Shanghai, China).

### *In vivo* tumor cell transplantation assay and drug treatments

For *in vivo* homing experiments, HCT116-Luc+ CRC cells were pretreated with CAF-CM, control medium, CAF-CM+C16:0 (50 μM), or CAF-CM+C18:1 (50 μM) for approximately 48h, and the cells (1×10^6^) were then collected and injected intraperitoneally into 6-week-old athymic nude female mice in 100 µL of PBS. Two weeks later, the mice were imaged using an IVIS Spectrum imaging system (Caliper Life Sciences, Hopkinton, MA).

To evaluate therapeutic effects in PM-CRC, mice intraperitoneal injected with HCT116 cell-derived (1×10^6^, i.p.) tumors one week after injection were treated with vehicle (p.o.), C16:0 (10 mg/kg once daily, p.o.), 5-FU (25 mg/kg twice a week, i.p.) or C16:0+ 5-FU (10 mg/kg once daily, p.o. + 25 mg/kg twice a week, i.p.) for 2 weeks (n=4 per group). After two weeks of treatment, the mice were imaged using an IVIS Spectrum imaging system (Caliper Life Sciences, Hopkinton, MA).

### Nontarget metabolomics analysis

The indicated cell culture supernatant was collected, and metabolites were extracted. Briefly, 50 μL of the cell culture supernatant was transferred to an EP tube. After the addition of 200 μL of extract solution (50% acetonitrile: 50% methanol, containing an isotopically labeled internal standard mixture), the samples were vortexed for 30 s, sonicated for 10 min in an ice-water bath, and incubated for 1 h at -40 °C to precipitate the proteins. Then, the sample was centrifuged at 12000 rpm for 15 min at 4 °C. The resulting supernatant was transferred to a fresh glass vial for analysis (Biotree Biomedical Technology Co., Ltd., Shanghai, China).

### Fatty acid uptake

HCT116 or DLD1 cells were treated with the indicated CAF-CM for 48 h. Cells were rinsed with PBS and incubated in DMEM (1% BSA) containing 2 μM C1-BODIPY 500/510 C12 (4,4-difluoro-5-methyl-4-bora-3a,4a-diaza-s-indacene-3-dodecanoic acid, Molecular Probes, Eugene, OR) for 30 min at 37 °C. Fatty acid uptake was analyzed by flow cytometry (Beckman Coulter, Miami, FL, USA).

### Use of stable isotopes to trace the fate of fatty acid derived by CAF

For ^13^C tracing analysis, CAFs treated with or without 10 μM Galunisertib (Selleck, Houston, TX, USA) were incubated with 20 uM [U-^13^C]-Stearic acid (CK Gas, Cambridgeshire, UK) for 24 h, then conditioned medium was collected. HCT116 cells were cultured in completed DMEM (50%) + indicated ^13^C-labeled CAF (Gal+)-CM or CAF (Gal-)-CM (50%) for 48 h. Then ^13^C-labeled fatty acids in HCT116 cells were determined. Briefly, cells (1×10^7^) were collected, washed twice with cold 0.9% saline, then 300 µL of methanol was added to the cells to quench the metabolic reactions, and 100µl of water was added. The cells were then collected by scraping and placed in 1.5-mL Eppendorf tubes, and 600 µL of chloroform was added to each tube. The cell extracts were vortexed at room temperature for 5 min and then centrifuged at 15,000 ×g for 5 min at 4°C. The upper layer (800 µL) was transferred to a new tube and dried by nitrogen gas flow for subsequent derivatization. The dried metabolites were dissolved by addition 500 µL of 2% (w/v) sulfate solution in methanol and incubated at 50 °C for 120 minutes. Samples were then added 100 µL of saturated sodium chloride solution and 500 µL of hexane, vortexed for 10s. The upper layer (450 µL) was transferred to a new tube. Another 500 µL of hexane was added, and the upper layer (450 µL) was collected. The total upper layer (900 µL) was dried by nitrogen gas flow. The derivatized products were dissolved in 100 µL of hexane and analyzed by GC-MS using the Thermo Fisher Trace 1310 with the 30m TM-35ms column (Thermo Fisher) connected to the Thermo Fisher IQS MS QD. The GC/MS was done at the Metabolic Innovation Center of Sun Yat-Sen University.

### Statistical analysis

Data were analyzed by Student's t test or one-way ANOVA using GraphPad Prism analytical software. The results are expressed as the mean ± SD, and a P value <0.05 was considered to indicate statistical significance.

## Results

### Lipidomics analysis revealed that phosphatidylcholine with unsaturated acyl chains was increased in PM-CRC patients

**Figure 1:**
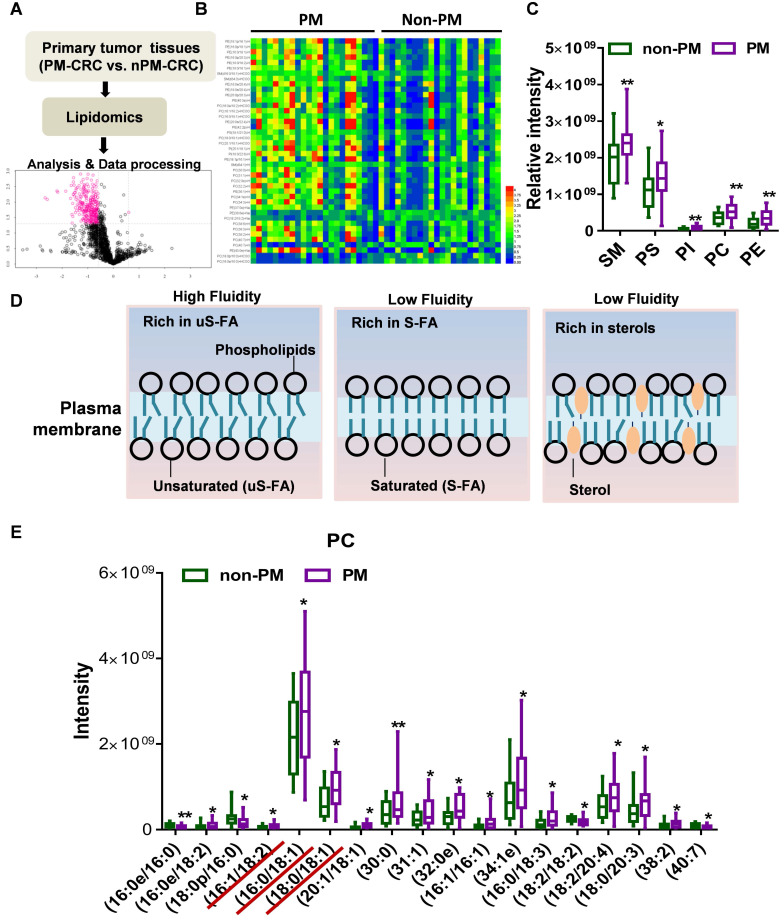
** Lipidomics analysis of the lipids whose abundance was significantly changed in clinical PM-CRC specimens. A,** Schematic of the lipidomics analysis of primary tumors from patients with nonperitoneal metastatic colorectal cancer (Non-PM, n=22) or peritoneal metastatic colorectal cancer (PM, n=23). **B,** Altered lipids are displayed as clusters in the heat map. **C,** Relative intensity of lipid species whose abundance was significantly changed. **D,** Lipid composition influences physical membrane properties. **E,** Distributions of phosphatidylcholine (PC) with two hydrophobic acyl chains within different lipid classes in the clinical CRC specimens. Bars, mean ± SD. *p < 0.05, **p < 0.01.

Using lipidomic analysis, we comprehensively identified and quantified the lipid profiles of clinical CRC specimens (primary tumors) obtained from a cohort of 45 patients (22 non-PM vs. 23 PM) (**Fig. 1A**). The abundance of a total of 42 lipid species was found to be significantly changed. Lipids whose abundance significantly differed between patients with and without PM are depicted in a heatmap in **Figure 1B (Table S1)**. A large percentage of the membrane's lipid matrix is made up of phospholipids 21. Interestingly, we found that the lipid species whose abundance was significantly different were mainly membrane lipids: phosphatidylcholine (PC), phosphatidylserine (PS), phosphatidylethanolamine (PE), phosphatidylinositol (PI) and sphingolipids (SMs) (**Fig. 1C, Fig. S1A**). PE, PS and PI are key determinants of membrane fusion and membrane surface charge and mediate the binding of peripheral membrane proteins. SMs usually affect the membrane packing density. Phospholipids constitute the bulk of the membrane's lipid matrix, and PC accounts for more than 50% of all phospholipids. PC usually contains an unsaturated acyl chain to increase membrane fluidity (**Fig. 1D**)^21^. Analyses of the metabolomic data from PC with unsaturated and saturated acyl chains among its two hydrophobic acyl chains were next performed. Primary tumors from patients with PM-CRC exhibited increased levels of PC with one (16:0e/18:2, 18:0/18:1, 16:0/18:1) or two unsaturated acyl chains (16:1/18:2, 20:1/18:1) **(Fig. 1E, Fig. S1B)** but decreased levels of PC with acyl chain saturation (16:0/16:0, 18:0/16:0) (**Fig. 1E, Fig. S1C**). PC contains at least one unsaturated acyl chain, such as 31:1, 38:2, 38:3, 34:1e, 38:6, 34:3, 32:2, and 40:7, which cannot be distinguished from the two acyl chains; such species were significantly increased in primary tumors from patients with PM-CRC **(Fig. 1E, Fig. S1B)**. The results showed that PC containing 30:0 and 32:0e was increased in the PM-CRC group, which was not unexpected. However, it is well known that fluidity is promoted by short uS-FAs 21. Consistently, relatively high amounts of shorter-chain FAs (C16-C18) and uS-FAs (16:1, 18:1, 18:2) was observed in the PM-CRC group, and these levels were higher than those in the group without PM (**Fig. 1E**). Notably, 16:0/18:1, the most abundant acyl chain of PC, was obviously upregulated (**Fig. 1E**). These results suggested that alterations in membrane lipids and unsaturated acyl chains may play a significant role in causing PM.

### PC with unsaturated acyl chains and membrane fluidity were increased by CAF-CM in CRC cells

**Figure 2:**
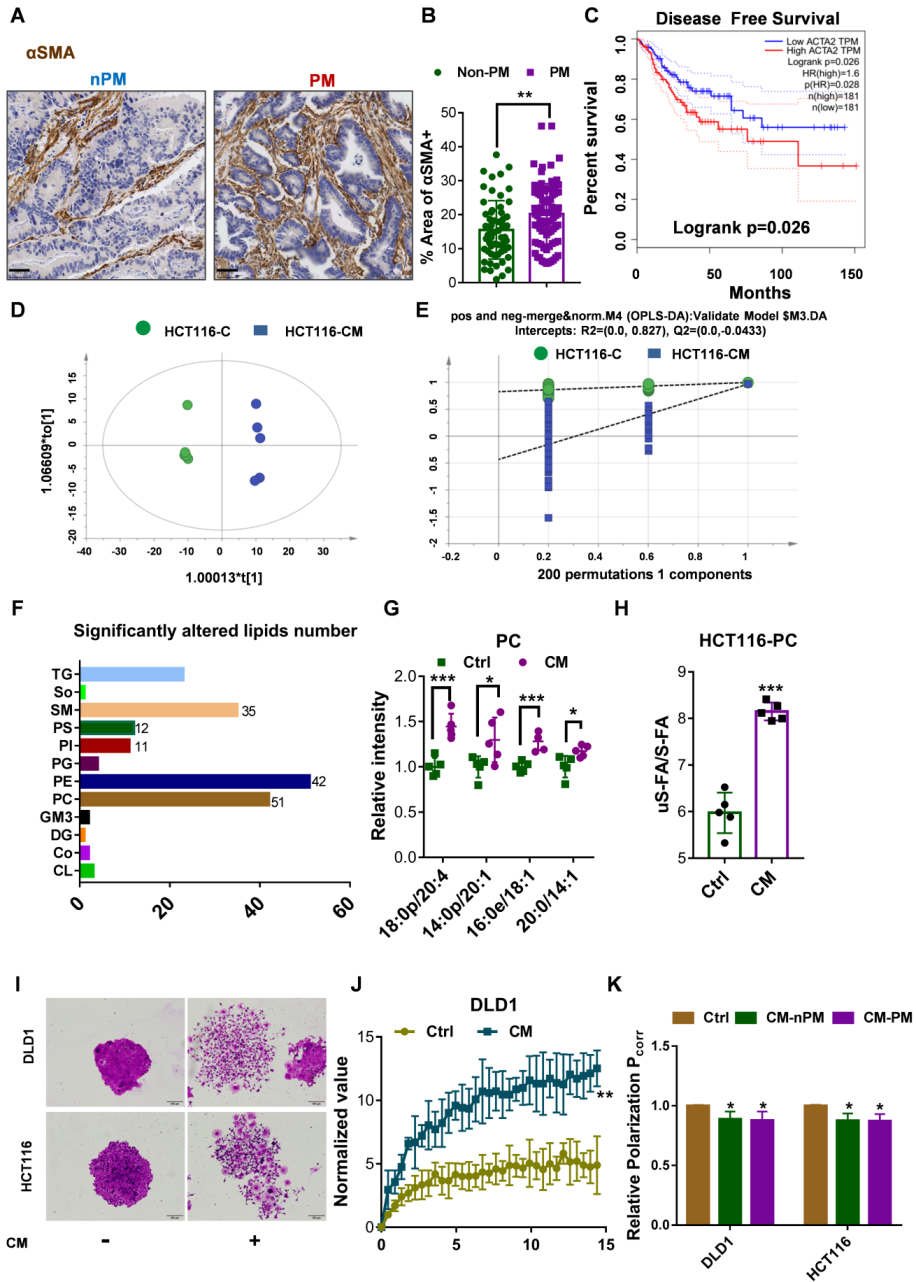
** CAFs increases unsaturated fatty acid chains and membrane fluidity in CRC cells**. **A,** Sections of non-PM and PM colorectal tumor tissue were stained for αSMA. Representative images from each group. Scale bars, 50 µm. **B,** Comparison of αSMA^+^ areas in non-PM (n=59) and PM (n=83) colorectal tumor tissue samples by IHC. **C,** Disease-Free Survival curves for colon and rectal adenocarcinoma patients were plotted with data from the TCGA database. The median cutoff point for αSMA (ACTA2) expression was used. There were 181 patients with low expression (the blue curve) and 181 with high expression (the red curve). **D,** Score plots are shown for the control (green) versus CAF-CM (blue) treated from the OPLS-DA model. n=5. **E,** Permutations are shown for the control (green) versus the CAF-CM-treated (blue) from the OPLS-DA model. n=5. **F,** Numbers of lipids whose abundance was significantly altered in CAF-CM-incubated HCT116 cells. n=5. **G,** Distributions of PC with unsaturated acyl chains in CAF-CM-incubated HCT116 cells. n=5. **H,** Ratios of unsaturated to saturated acyl chains in CAF-CM-incubated HCT116 cells. n=5.** I,** Clonogenic formation in CRC cells with or without CAF-CM incubation. After approximately 14 days of incubation, the colonies were stained with crystal violet and then imaged using an inverted microscope. Representative data are shown.** J,** Normalized fluorescence recovery curve of DLD1 cultured with control/conditioned medium. n = 3. **K,** Fluorescence polarization in CRC cells incubated in CAF-CM. n =3. Bars, mean ± SD. *p < 0.05, **p < 0.01.

CAFs are the most abundant stromal cells in the microenvironment of solid tumors. αSMA is a common marker of CAFs that is highly expressed in the human colonic circular muscle layer, pericryptal fibroblasts and pericytes but not in normal epithelial cells, mesenchymal fibroblasts or cancer cells^22^-^24^. Our data indeed showed that αSMA^+^ CAFs were the prominent component in patients with CRC of high-grade pathology (stage T4Nx) by IHC (**Fig. 2A**). Furthermore, in primary PM-CRC tumors, αSMA^+^ CAFs were present at a higher relative abundance (a higher proportion of the αSMA^+^ area) (**Fig. 2B**), and high αSMA^+^ (ACTA2) expression was associated with poor prognosis in CRC patients based on analysis of data from the TCGA database (**Fig. 2C**).

In our previously published research, we showed that isolated CRC CAFs contribute to CRC cell migration, invasion and intraperitoneal dissemination by enhancing FA catabolism (**Fig. S2A**) 16. To further evaluate whether CAF induced the increase in PC with an unsaturated acyl chain, lipidomics analysis was further used to comprehensively identify and quantify the lipid compositions of HCT116 cells after incubation with CAF-CM. A total of 1,790 unique lipids from 35 lipid classes were identified by integrating positive and negative electrospray ionization tandem MS data. Principal coordinate analysis (PCA) and the orthogonal partial least squares discriminate analysis (OPLS-DA) model revealed major differences between control medium and CAF-CM-treated cells (**Fig. 2D-E**). Employing the OPLS-DA method and univariate statistical analysis, the metabolites whose abundance was significant different were screened with the OPLS-DA model using a VIP >1 and a P value <0.05 as the screening criteria, and a total of 187 lipid species were found to be significantly changed in abundance, among which 48 metabolites were downregulated, and 139 were upregulated (**Fig. 2F, Fig. S2B, Table S2**). Interestingly, the main types of membrane lipids (PC, PM, PI, SM, PE) accounted for the vast majority of the total lipid species whose abundance was significantly changed (**Fig. 2F**). Further analysis showed that the levels of unsaturated fatty acyl chain-containing PC, including 18:0p/20:4, 14:0p/20:1, 16:0p/18:1, 20:0/14:1, in CAF-CM-incubated HCT116 cells were significantly increased (**Fig. 2G**). However, no significant difference in cholesterol (ChE) content was found (**Fig. S2C**). In addition, PC was enriched in unsaturated fatty acyl chains but lower in saturated fatty acyl chains in cells after incubation with CAF-CM (**Fig. S2D**). Notably, the ratio of unsaturated versus saturated fatty acyl chains in PC in HCT116 cells was clearly increased after CAF-CM treatment (**Fig. 2H**).

The lipid composition of cellular membranes can influence physical properties and is regulated to maintain membrane fluidity 25. The balance between S-FAs and uS-FAs is a key factor in determining membrane fluidity 26, 27. PC usually contains an unsaturated acyl chain, which increases membrane fluidity, as shown in **Figure 1D**. Based on the results of lipidomics analysis, we postulated that CAFs promote the intraperitoneal dissemination and invasion of CRC cells by increasing cell membrane fluidity. First, crystal violet staining was used to observe morphological changes to evaluate the relationship between membrane fluidity and CRC CAFs. As shown in **Figure 2I**, CAF-CM treatment altered cell morphology and promoted motility during colony formation. The invasion and motility of tumor cells can be promoted by increased membrane fluidity; therefore, we next evaluated the membrane fluidity of CRC cells after CAF-CM incubation. FRAP was applied to detect the plasma membrane fluidity. The fluorescence recovery rate in the bleached area of cells was calculated, and curves showed the intensity of recovered fluorescence of the bleached membrane areas. Specifically, the fluorescence recovery rate in cells cultured with CAF-CM was significantly higher than that of cells cultured with the control medium (**Fig. 2J, Fig. S2E**). Membrane fluidity is widely studied by the fluorescence polarization technique. Generally, higher polarization values correspond to lower membrane fluidity 19. Indeed, polarization, as determined by TMA-DPH, was also decreased in CRC cells after incubation with CAF-CM (**Fig. 2K**). All these findings indicated an increase in membrane fluidity. Interestingly, no difference was found in the membrane fluidity of CRC cells incubated with CAF-CM from patients with CRC with or without PM (Fig. 2K). However, CAF-CM dose-dependently increased membrane fluidity, as shown by a decrease in relative fluorescence polarization (**Fig. S2F**), consistent with the observation that CAFs were present at higher relative abundance in primary PM-CRC tumors. Overall, these findings demonstrated that colorectal CAFs can induce an increase in unsaturated fatty acyl chain-containing PC that is accompanied by increased membrane fluidity.

### C16:0 decreases membrane fluidity and prevents migration, invasion and intraperitoneal dissemination

**Figure 3:**
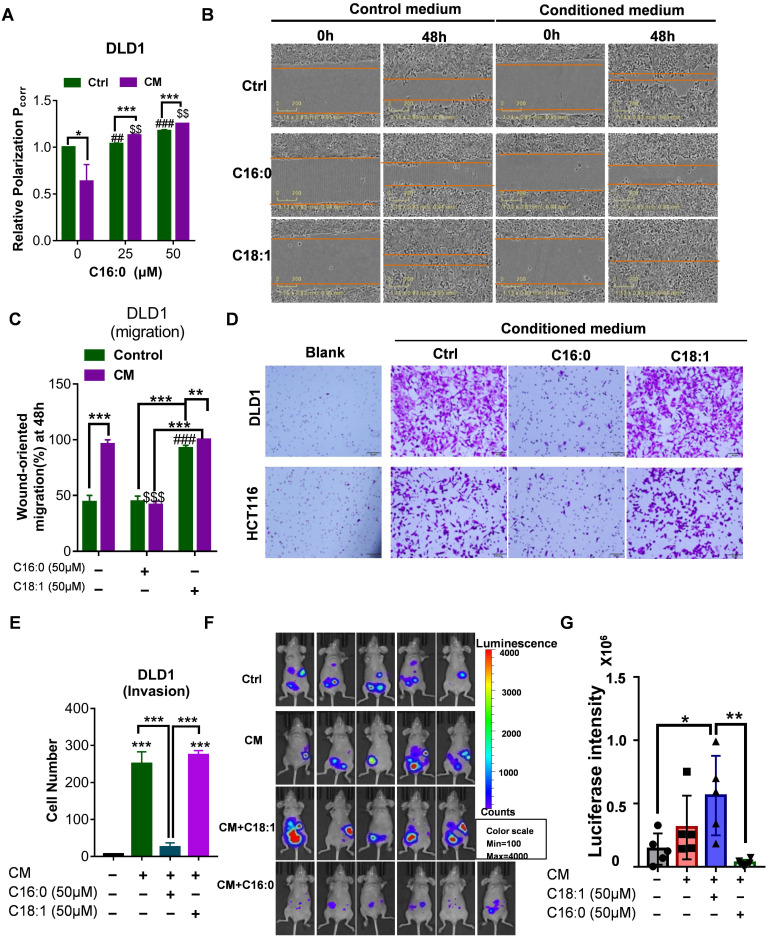
** C16:0 inhibits CRC cell migration, invasion and intraperitoneal dissemination by decreasing membrane fluidity. A,** Fluorescence polarization of CAF-CM/C16:0-treated DLD1 cells. #, $ indicates comparisons with the corresponding control groups. n=3. **B,** Wound healing assay of DLD1 cells incubated with CAF-CM and treated with the indicated compounds as detected by IncuCyte ZOOM. n=3. **C,** Quantification of the wounding rates of DLD1 cells in Fig. 3B. #, $ indicates comparisons with the corresponding control groups. Wound width was normalized to the initial width when the wound was created. n=3. **D,** Crystal violet staining was used to quantify DLD1/HCT116 cell Transwell invasion after 24 h of exposure to CAF-CM and treatment with the indicated compounds. n=3. **E,** The number of crystal violet-stained cells among DLD1 cells that invaded Transwell membranes is shown in Fig. 3D. n=3. **F,** Animals from each test group were bioimaged (Xenogen IVIS system) by detecting the luciferase emission spectrum at 2 weeks after i.p. injection to visualize the progression of tumor growth in the peritoneum. n=5 or 6. **G,** The luciferase intensity of each test group was analyzed. Bars, mean ± SD. *p < 0.05; ##, $$, **p < 0.01; ###, $$$, ***p < 0.001.

Lipids containing S-FAs are known to decrease fluidity. Based on the finding of Lin et al. that saturated C16:0 supplementation could decrease cell membrane fluidity and inhibit tumor cell migration and invasion 17, we hypothesized that a S-FA (C16:0, palmitic acid) could inhibit CRC cell migration and invasion induced by CAFs. First, we showed that C16:0 supplementation decreased cell membrane fluidity in a dose-dependent manner (**Fig. 3A, Fig. S3A**). Indeed, exposure of DLD1 or HCT116 cells to C16:0 severely suppressed migration (**Fig. 3B-C, Fig. S3B-C**). CRC cells incubated with CAF-CM also exhibited decreased migration compared to that of CRC cells incubated with another S-FA (C18:0, stearic acid) and increased migration compared to that of CRC cells incubated with an uS-FA (C16:1, methyl palmitoleate; C18:1, oleic acid, OA) (**Fig. S4**). Moreover, the number of invaded DLD1 and HCT116 cells treated with C16:0 was significantly reduced (**Fig. 3D-E, Fig. S3D**), suggesting that C16:0 treatment inhibited cell migration and invasion *in vitro*. Then, we investigated whether C16:0 could inhibit the peritoneal dissemination of CRC cells in nude mice following intraperitoneal injection of human HCT116 cells. Intraperitoneal tumor growth was visible in all mice in the peritoneal dissemination model, while the luciferase intensity of the CAF-CM-treated group was stronger than that of the control group, even though no statistical significance was reached. As shown in **Figure 3F and G**, intraperitoneal tumor growth in the C16:0 treatment group was much lower than that in the control group. As expected, the monounsaturated FA (MUFA) C18:1 significantly increased tumor migration, invasion and intraperitoneal dissemination. These results suggested that C16:0 can attenuate CRC cell invasion, and intraperitoneal dissemination induced by CAFs may occur through decreased cell membrane fluidity.

### Increased cell membrane fluidity enhances glucose uptake and metabolism

**Figure 4:**
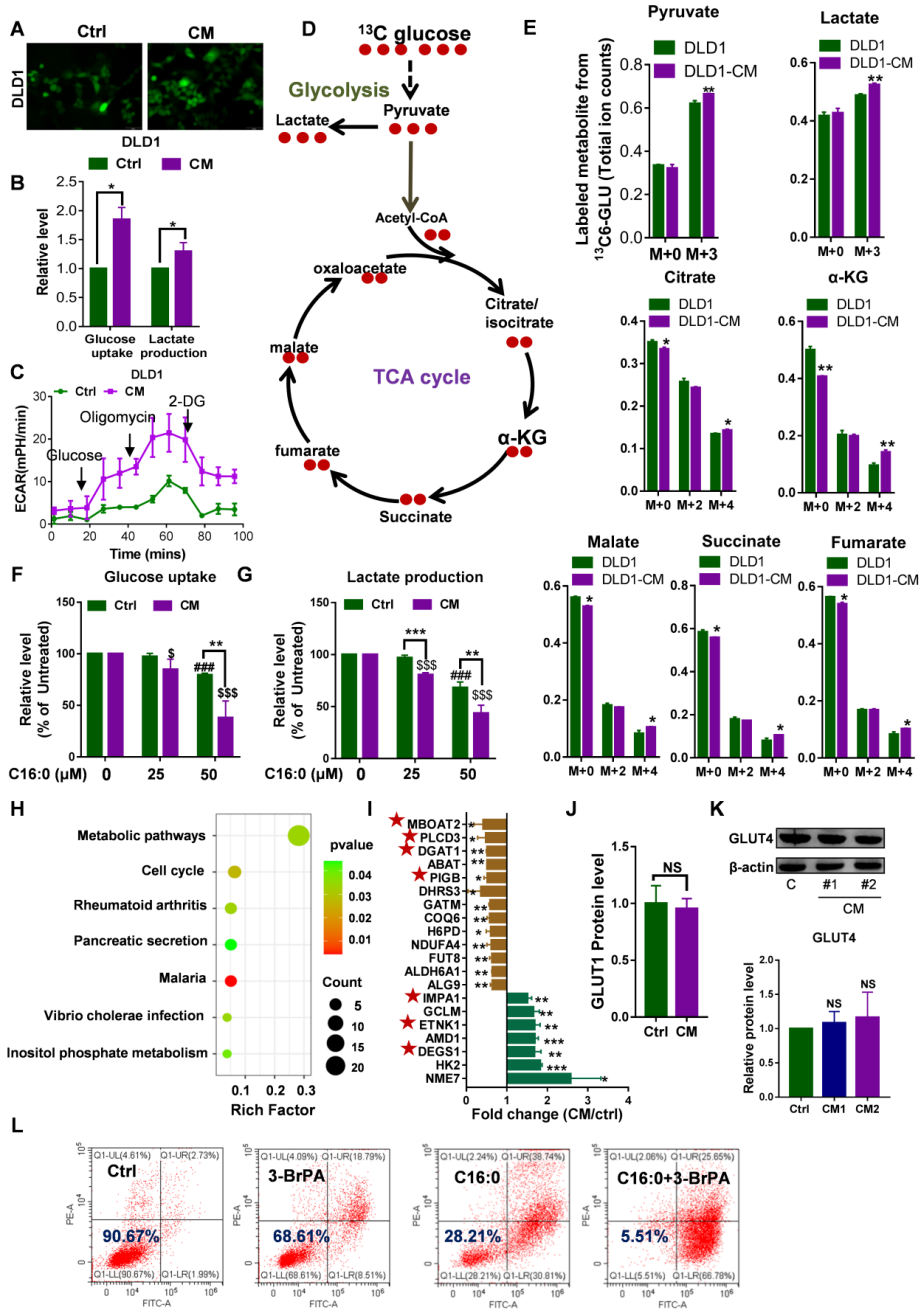
** Increased membrane fluidity induced by CAFs can accelerate glucose metabolism. A,** Glucose uptake in DLD1 cells incubated with CAF-CM or control medium visualized by 2-NBDG fluorescence. n=3. **B,** Relative levels (% of control) of glucose uptake and lactate production in DLD1 cells after incubation with CAF-CM. n=3. **C,** Impact of CAF-CM incubation on the extracellular acidification rate (ECAR, an indicator of lactate production from glycolytic metabolism) as determined by Seahorse analyzer. n = 2 independent experiments with similar results. **D,** Schematic illustration of glucose recycling using U-^13^C-glucose and tracing analysis. DLD1 cells were incubated with or without CAF-CM for 48 h. Then, cells were cultured in U-^13^C-glucose-containing DMEM for 24 h to synthesize U-^13^C metabolites. **E,** U-^13^C metabolites were analyzed. n=3. **F,** Glucose uptake and **G,** lactate production in CAF-CM-treated DLD1 cells cultured in the absence or presence of C16:0 (concentration from 0 to 50 µM). n=3. **H,** The metabolic pathway was highly enriched in CAF-CM-treated DLD1 cells by proteomics analysis. All pathways displayed had a P value < 0.05. **I,** Fold changes in the expression of metabolic pathway proteins determined by proteomics analysis. Brown bars indicate downregulated proteins; green bars indicate upregulated proteins. Those proteins labeled with a star are involved in phospholipids and sphingolipid metabolism. **J,** The protein level of GLUT1 in CAF-CM-treated DLD1 cells determined by proteomics analysis. n=3. **K,** The protein level of GLUT4 in CAF-CM-treated DLD1 cells determined by Western blotting. n = 3. **L,** The viability of DLD1 cells incubated with CAF-CM after 48 h of incubation with 3-BrPA was assessed by annexin-V/PI assay. The value in each panel indicates the % of surviving cells. n = 2 independent experiments with similar results. Bars, mean ± SD. *p < 0.05, **p < 0.01.

Increased membrane fluidity is widely accepted to facilitate membrane fusion and influence the binding and activity of peripheral membrane proteins 21. Glucose uptake experiments were carried out to quantify an important membrane function: a very strong 2-NBDG signal was detected by fluorescent staining, and glucose uptake and lactate production were significantly increased in CAF-CM-incubated CRC cells, as detected with a YSI 2950 STAT Plus instrument (**Fig. 4A-B, Fig. S5A-B**). Additionally, analysis of the ECAR showed that CAF-CM-treated CRC cells exhibited increased glycolytic metabolism (**Fig. 4C, Fig. S5C**). Consistent with these results, glycolytic flux analysis by [U-^13^C] glucose tracing provided evidence that CAF-CM-incubated CRC cells also exhibited greater flux through the glycolytic pathway (**Fig. 4D-E**). Together, these data suggested that the rate of glucose metabolism in CAF-CM-incubated cells was increased. Importantly, increasing the C16:0 concentration reduced cellular glucose uptake and lactate production in a dose-dependent manner (**Fig. 4F-G, Fig. S5D-E**), accompanied by decreased membrane fluidity (**Fig. 3A, Fig. S3A**).

Label-free proteomic analysis revealed the greater metabolic-specific protein changes in DLD1 cells following CAF-CM treatment (**Fig. 4H**). Moreover, 7 of the 20 abnormally expressed proteins are involved in phospholipids and sphingolipid metabolism (**Fig. 4I**). The proteomics data revealed that expression of the glucose transporter GLUT1 was not obviously changed (**Fig. 4J**). Next, we performed immunoblot analysis of another tumor-related glucose transporter, GLUT4, in CAF-CM-incubated CRC cells. Western blot analysis identified no changes in GLUT4 in CAF-CM-incubated CRC cells (**Fig. 4K, Fig. S5F**). These results suggested that the enhanced glucose uptake cannot be attributed to upregulated glucose transporters but rather is due to increased membrane fluidity. Consistent with the increase in glycolysis, CAF-CM-incubated CRC cells contained significantly increased levels of the key mediator of aerobic glycolysis HK2. Indeed, the glycolysis inhibitor 3-BrPA acted synergistically with C16:0 to induce cell death in CRC cells treated with CAF-CM (**Fig. 4L, Fig. S5G**), indicating that reduced cell membrane fluidity and diminished glucose metabolism contribute to cell apoptosis and death.

### Lower SCD expression was associated with poor prognosis and PM in CRC patients

**Figure 5:**
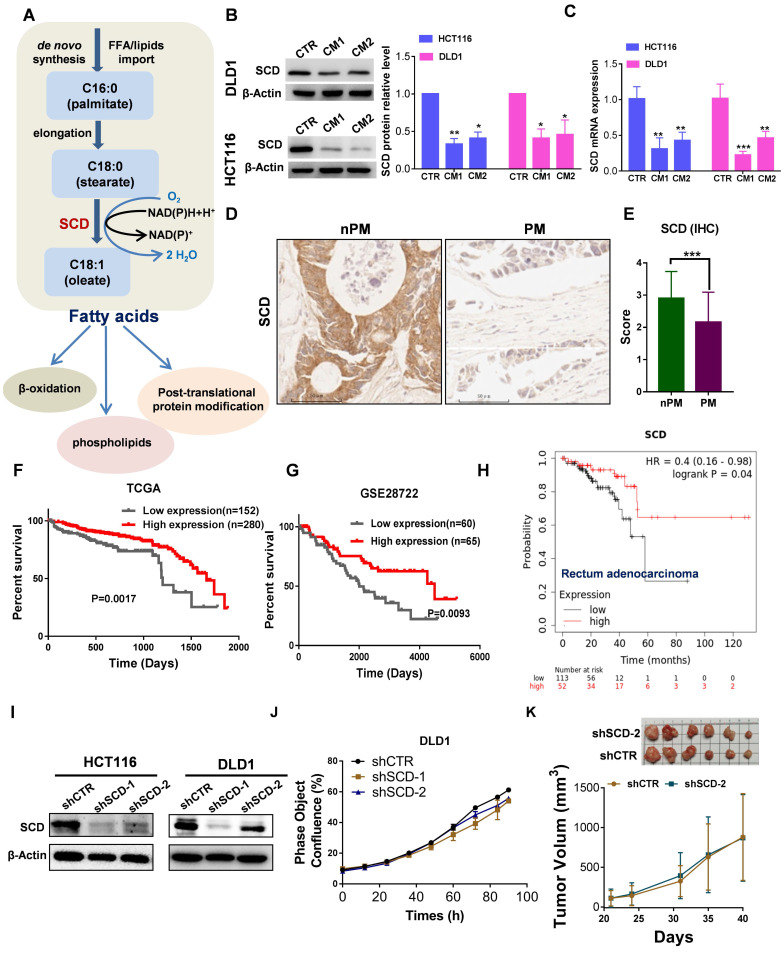
** Low expression of SCD is associated with poor prognosis in patients with CRC and PM-CRC. A,** Schematic representation of fatty acid synthesis and function. **B,** Western blot showing SCD expression in DLD1 and HCT116 cells incubated with CAF-CM. **C,** RT-qPCR was performed to measure the mRNA level of SCD in DLD1/HCT116 cells incubated with CAF-CM. D-E, Comparison of SCD protein expression by IHC (**D**). Magnification, ×200. Representative images from each group are shown. Staining scores for SCD in non-PM (n = 70) and PM (n = 56) tissues (**E**). **F,** Kaplan-Meier curves showing the 5-year survival rate of CRC patients from TCGA cohorts partitioned by the relative abundance of SCD (high/low expression). **G,** Kaplan-Meier curves showing overall survival of CRC patients from the GEO (GSE28722) cohort partitioned by the relative abundance of SCD (high/low expression). **H,** Survival curves were plotted for rectal adenocarcinoma patients (n = 165) using the Kaplan-Meier Plotter Database. The software autoselected the best cutoff point for SCD expression. There were 113 patients with low expression (the black curve) and 52 with high expression (the red curve). HR, hazard ratio. **I,** Western blot showing the efficiency of SCD knockdown. Anti-SCD antibody (1:1000 dilution; Affinity Biosciences, OH, USA). **J,** The proliferation rate of DLD1^low-SCD^ cells evaluated by phase object confluence (%) with IncuCyte ZOOM. n=3. **K,** The shCTR- or shSCD-2-transfected HCT116 cells (3.5*10^6 per mouse) were subcutaneously inoculated into Balb/C nude mice. Upon termination of the experiment, the tumors were isolated and photographed, n=6. Bars, mean ± SD. *p < 0.05, **p < 0.01, ***p < 0.001.

A proper ratio of uS-FAs to S-FAs contributes to regulating membrane fluidity. The balance between S-FAs and monounsaturated FAs (MUFAs) can be altered by SCD , also named delta-9 desaturase, which generates de novo MUFAs such as palmitoleic acid (16:1) and OA (18:1), major components of the plasma membrane (**Fig. 5A**). Angelucci et al. showed an increase in cancer cell motility and membrane fluidity induced by treatment with CAF-CM through the upregulation of SCD 28. However, after CRC cells were exposed to CAF-CM, the opposite was observed. As shown in **Figure 5B**, the protein expression of SCD significantly decreased, and a decrease in SCD expression was also observed at the mRNA level (**Fig. 5C**). Importantly, low expression of SCD was also found in PM-CRC tissues (**Fig. 5D-E**). Furthermore, analysis of public databases revealed that high-risk CRC patients express low levels of SCD and exhibit worse overall survival than low-risk CRC patients who had better survival and higher SCD expression (**Fig. 5F-H**). These results indicated that lower SCD expression in CRC is associated with significantly worse overall survival in patients. However, SCD is overexpressed in multiple human cancers, and SCD upregulation can increase cancer cell motility and membrane fluidity and promote metastasis and invasion 28, 29, which appears to be in conflict with our finding. We created stable cell lines to assess whether low SCD expression affects cell survival and proliferation in CRC cell lines (**Fig. 5I**). As shown in **Figure 5l-J and Figure S6A**, the proliferation rate of CRC^low SCD^ cells was slightly decreased *in vitro*. However, the downregulation of SCD did not cause an obvious decrease in tumor growth *in vivo* (**Fig. 5K**). Although SCD expression was low in CRC-PM tissues and CAF-treated HCT116 and DLD1 CRC cells, the ratio of uS-FAs to S-FAS in the membrane was still increased. One possible explanation is that the uptake of exogenous MUFAs or lipid-like molecules can compensate for the SCD deficit in CRC^low SCD^ cells.

### The uptake of CAF-derived lipids compensates for low SCD expression and boosts CRC growth and metastasis

**Figure 6:**
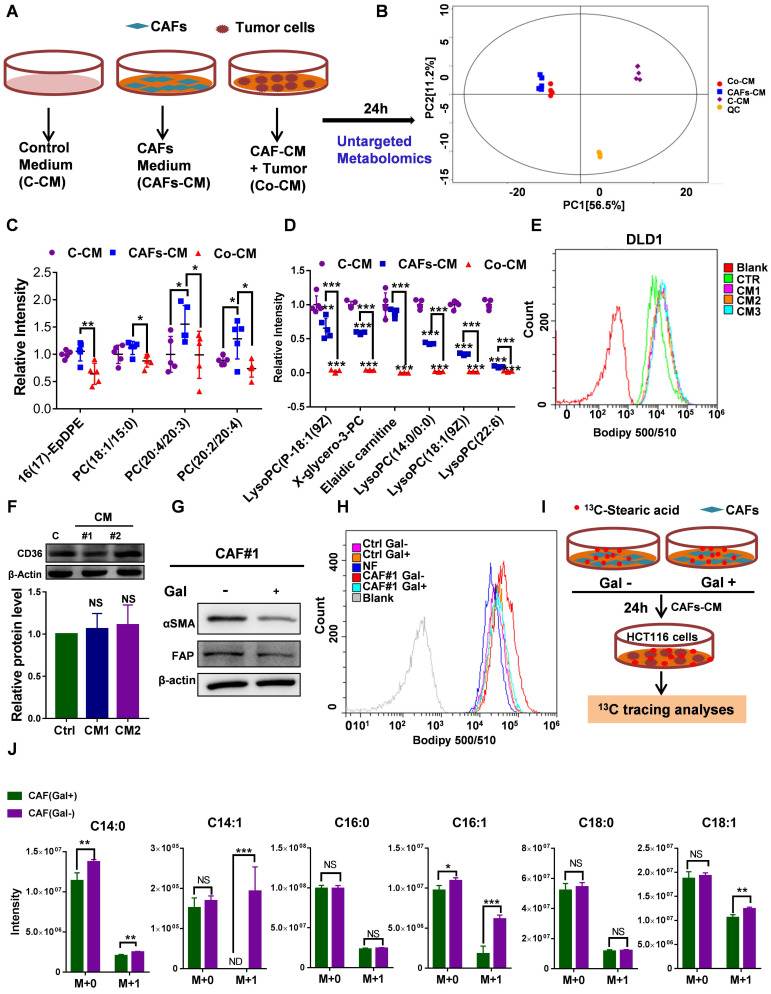
** Uptake of CAF-derived lipids by CRC through increased membrane fluidity. A,** An experimental schematic of the method used to collect the supernatant for nontarget metabolomics analysis. **B,** Score plots are shown for control medium (purple) versus CAFs-CM (blue) and CAF-CM+ HCT116 cells (Co-CM) (red) treated from the OPLS-DA model. **C,** Relative intensity of significantly increased lipid molecules secreted by CAFs but consumed by HCT116 cells, n=5. **D,** Relative intensity of significantly decreased lipid molecules in HCT116 cells incubated with the supernatant of CAFs-CM, n=5. **E,** The indicated treated CRC cells were incubated with BODIPY 500/510 to analyze the lipid uptake by flow cytometry. **F,** Western blot showing CD36 expression in DLD1 cells incubated with CAF-CM. Anti-CD36 antibody (1:1000 dilution; Affinity Biosciences, OH, USA). **G,** Western blot showing αSMA and FAP expression in CAFs treated with Galunisertib (10 µM). Anti-αSMA antibody (1:1000 dilution; Cell Signaling Technologies, MA, USA); Anti-FAP antibody (1:1000 dilution; Abcam, Cambridge, MA, USA). **H,** The DLD1 cells treated with indicated CM were incubated with BODIPY 500/510 to analyze lipid uptake by flow cytometry. **I,** An experimental schematic of the method used to collect the supernatant for fatty acid tracing analyses (U-^13^C- stearic acid). **J,** ^13^C labeled fatty acids in HCT116 cells were analyzed. n=4. Bars, mean ± SD. *p < 0.05, **p < 0.01, NS: not statistically significant, ND: not detected.

**Figure 7:**
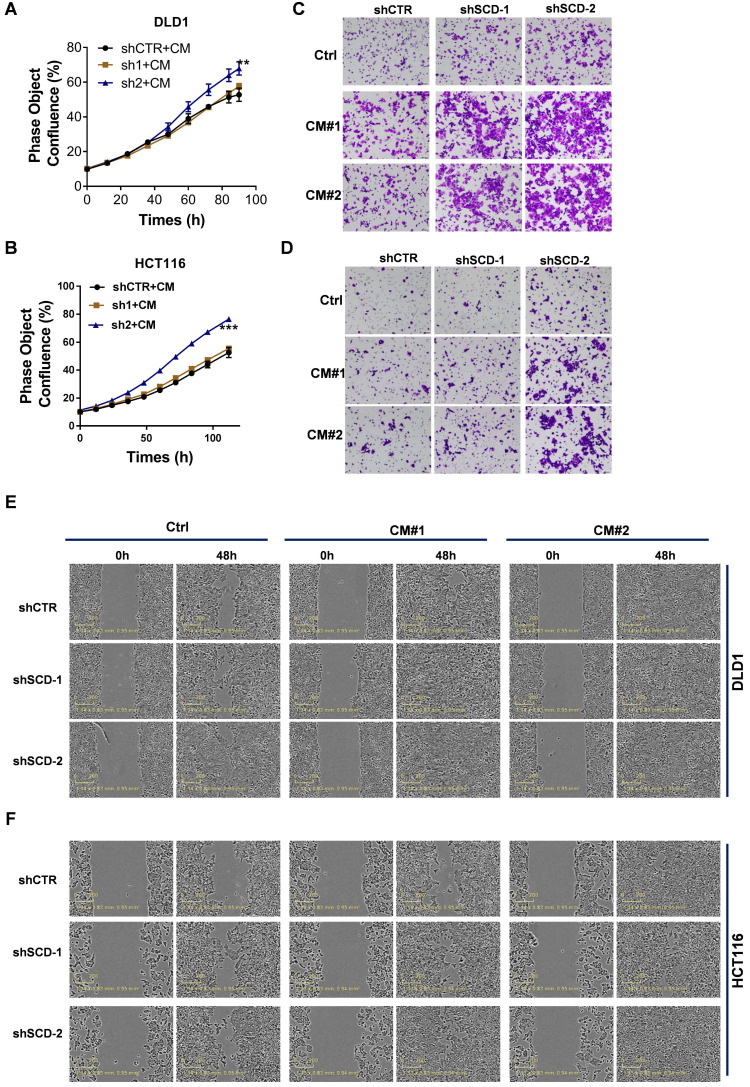
** CAF-derived lipids boosted CRC growth and metastasis. A-B,** The proliferation rate of DLD1^low-SCD^ (A) and HCT116^low-SCD^ (B) cells after incubation with CAF-CM was evaluated by phase object confluence (%) with IncuCyte ZOOM. n=3. **C-D,** Crystal violet staining was used to quantify the Transwell invasion of indicated DLD1 (C) and HCT116 (D) cells after 24 h of exposure to CAF-CM. **E-F,** Wound healing assay of the indicated DLD1 (E) and HCT116 (F) cells incubated with CAF-CM as detected by IncuCyte ZOOM. Bars, mean ± SD. *p < 0.05, **p < 0.01.

To explore whether CAFs secrete an abundance of lipids that support metabolism in CRC^low SCD^ cells, we detected differentially expressed metabolites in CM using nontargeted metabolomics analysis (**Fig. 6A**). PCA showed that the metabolites from CAF-CM and Co-CM clustered together, indicating that the metabolites from CAF-CM and Co-CM were significantly different from those from the control medium (**Fig. 6B**). Moreover, we determined the relative levels of all lipids and lipid-like molecules in the indicated CM (**Table S3**). Four lipid molecules were increased in the CAF supernatant compared to the control medium but significantly decreased after coculture with tumor cells (**Fig. 6C**). The data suggested that these lipid molecules secreted by CAFs were consumed by CRC cells. Our data showed that other lipids/lipid-like molecules, which may be from FBS, were greatly depleted in Co-CM, medium from HCT116 cells cocultured with CAF-CM (**Fig. 6D**). Increased lipid uptake can modify the plasma membrane, resulting in enhanced membrane fluidity. Supporting this notion, increased free FA (FFA) uptake after CAF-CM incubation was found (**Fig. 6E, Fig. S6B**). Notably, FA transporter levels in CAF-CM-incubated CRC cells were not obviously changed (**Fig. 6F, Fig. S6C**). Given that TGF-β plays an important role in fibroblast activation 30, we asked whether TGF-β pathway blockade could be attributed to inhibiting effects on tumor cells induced by CAFs. We used the TGFBR1 specific inhibitor Galunisertib, which was investigated in a phase 2 trial to treat HCC, a small-molecule cancer drug that specifically inhibits TGF-beta signaling 31, 32. Consistent with a role for TGF-β pathway activation in fibroblasts, inhibition of TGF-β reduced the makers of fibroblast activation, as evident by a decrease in the protein level of FAP and α-SMA (**Fig. 6G**). Blocking fibroblast activation led to a significant inhibition in increasing FFAs uptakes (**Fig. 6H**). Meanwhile, the result also showed that NF-CM does not appear to have favorable effects on lipid uptake (**Fig. 6H**). To assess the fate of CAF-derived lipids or fatty acids, CRC CAFs were treated with or without Galunisertib (10 uM) in a medium containing U-^13^C-Stearic acid for 24h. CM was collected and then treated HCT116 cells (**Fig. 6I**). Using ^13^C tracing analyses, we showed that ^13^C labeled metabolites, C14:0 (M+1), C14:1 (M+1), C16:1 (M+1), C18:1 (M+1), were significantly higher in CAFs (Gal-) CM treated HCT116 cells (**Fig. 6J, Table S4**). In addition, intracellular unlabeled fatty acids, C14:0 (M+0) and C16:1 (M+0), were also higher in HCT116 cells cultured with CAF (Gal-) CM, indicating an increased FA availability. Importantly, the slight decrease in the proliferation of CRC^low SCD^ cells was reversed in the CAF-CM supplementation group (**Fig. 7A-B**). Lipid/lipid-like molecule uptake can modify membrane fluidity, which is closely related to cell motility. Then, we found that CAF-CM could increase CRC^low SCD^ cell migration in both wound healing and Transwell migration assays (**Fig. 7C-F**). Taken together, these results demonstrated that CAF-derived lipids or exogenous lipids could compensate for low SCD expression and then alter lipid composition, further influencing cell membrane fluidity, which plays a profound role in promoting tumor growth and metastasis.

### High concentrations of C16:0 induced lipotoxicity and cell death in CAF-CM-treated CRC cells

**Figure 8:**
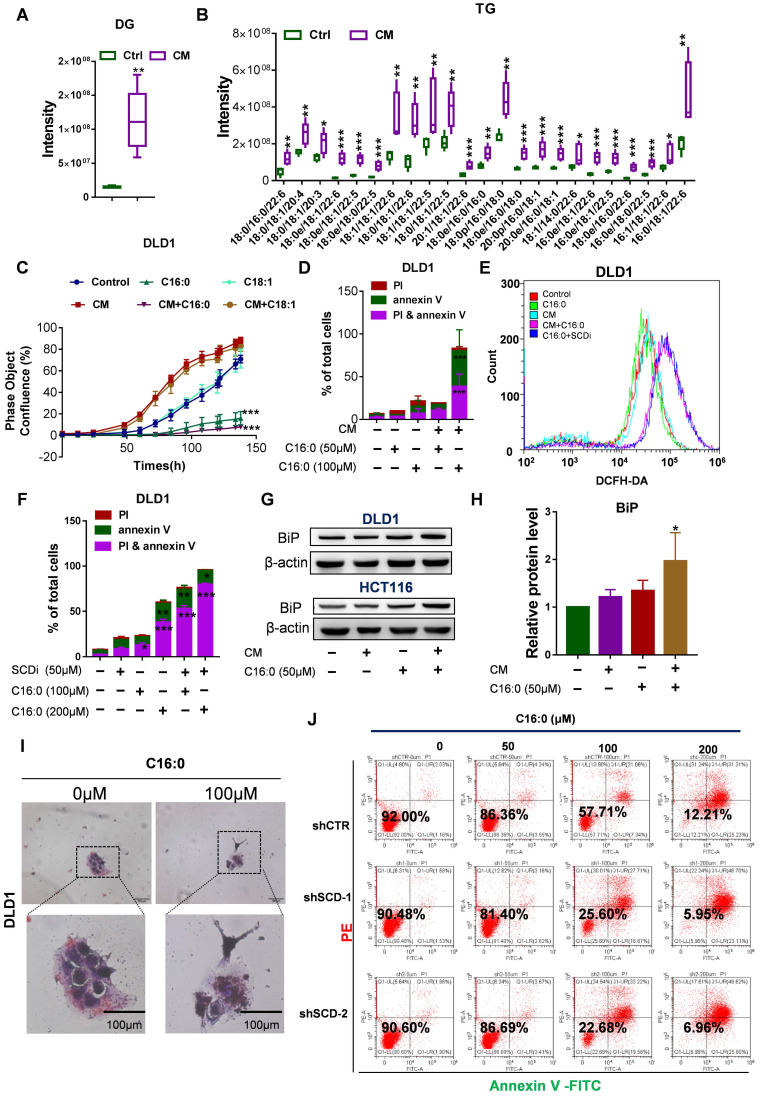
** High doses of palmitate aggravated the lipotoxicity of CAF-CM- treated CRC cells. A-B,** Quantification of diacylglycerol (DG; A) and triacylglycerol (TG; B) by lipidomics. **C,** Growth curves of DLD1 cells treated with the indicated compounds. IncuCyte ZOOM software was used to process the image data for phase object confluence. n=3. **D,** C16:0 induced apoptotic cell death in DLD1 cells. Cell viability assay of DLD1 cells treated with different concentrations of C16:0 (0, 50 and 100 µM) for 48 h and then detected by the annexin-V/PI assay. Apoptotic cells were identified by propidium iodide (PI) and annexin V staining. n=3. **E,** Reactive oxygen species (ROS) levels in DLD1 cells with or without CAF-CM incubation and SCD inhibitor treatment were detected by flow cytometry. n = 3. **F,** SCD inhibitor treatment increased cellular sensitivity to C16:0 in DLD1 cells. Cell viability was assessed by annexin-V/PI assay. n=3. **G,** BIP (GRP78 BiP) protein levels in DLD1/HCT116 cells incubated with CAF-CM or after C16:0 treatment (50 µM, ~6 h) was detected by Western blotting. Anti-BIP antibody (1:1000 dilution; Abcam, Cambridge, MA, USA), n=3. **H,** Relative protein level of BIP in DLD1 cells. **I,** DLD1 cells incubated with CAF-CM and then treated with C16:0 were detected by oil red/hematoxylin staining. The cells were imaged using an inverted microscope. n = 3. **J,** Cell viability assay of the indicated DLD1 cells treated with different concentrations of C16:0 (0, 50, 100 and 200 µM) for 48 h and then detected by the annexin-V/PI assay. Apoptotic cells were identified by propidium iodide (PI) and annexin V staining. n=3. Bars, mean ± SD. *p < 0.05, **p < 0.01, ***p < 0.001.

Piccolis et al. showed that treatment of cells with palmitate increased saturated glycerolipids and ER stress 33. In comparison to cells cultured in control medium, CAF-CM-treated cells exhibited large increases in the relative amounts of diacylglycerol (DG) and triacylglycerol (TG) (**Fig. 8A-B**). Therefore, we suspected that CAF-CM-treated cells would be more intolerant to high palmitate concentrations due to lipotoxicity, which prompted us to determine whether high-dose S-FA (C16:0) treatment could induce CRC cell apoptosis. As expected, the inhibitory effect of exogenous C16:0 on CRC cell growth was time-dependent (**Fig. 8C, Fig. S7A**). Moreover, we performed an annexin V/PI apoptosis assay after the cultures were exposed to C16:0 for 48 h and found a significant increase in the percentage of apoptotic cells among the CAF-CM-incubated cells (**Fig. 8D, Fig. S7B and G**). However, an uS-FA (C18:1) had no inhibitory effect in either cell line pretreated with CAF-CM (**Fig. 8C, Fig. S7A**).

Increased C16:0 concentrations induce apoptosis. To identify the mechanisms by which C16:0 induces cellular lipotoxicity, we measured the reactive oxygen species (ROS) content of the indicated cells after treatment with C16:0. To eliminate the excessive ROS whose formation was induced by apoptosis, ROS were detected after a 5-h treatment period with the indicated compounds. Here, we found that the SCD inhibitor PluriSIn #1 and C16:0, as well as CAF-CM treatment and C16:0, synergistically enhanced ROS production (**Fig. 8E and Fig. S7C**). Indeed, direct exposure of CRC cells to C16:0 in the presence of the SCD inhibitor PluriSIn #1 significantly increased C16:0-induced cell death (**Fig. 8F, Fig. S7D and H**). Moreover, immunoblotting with a specific antibody against GRP78 BiP (BiP) demonstrated that CAF-CM-incubated cells exhibited strongly enhanced endoplasmic reticulum (ER) stress (also known as the unfolded protein response [UPR]) after C16:0 treatment (**Fig. 8G-H, Fig. S7E**). As suggested by previous studies 34-36, we found that CAF-CM-treated cells had elevated lipid accumulation after treatment with C16:0, which further promoted lipotoxicity-induced CRC cell apoptosis (**Fig. 8I, Fig. S7F**). Moreover, knockdown of SCD in CRC cells significantly increased their sensitivity to C16:0. This result is consistent with the increased abundance of apoptotic cells among the CAF-CM-incubated CRC cells treated with C16:0 (**Fig. 8J, Fig. S8**).

### C16:0 and 5-FU synergistically and effectively inhibit CAF-induced CRC cell growth and peritoneal seeding

**Figure 9:**
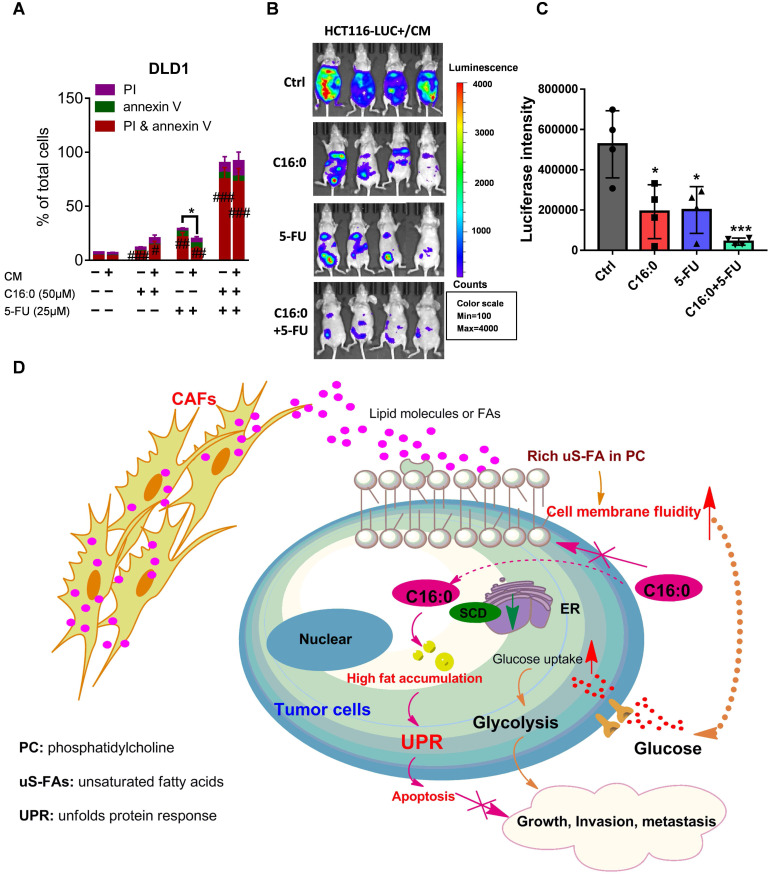
** C16:0 acts synergistically with 5-FU to inhibit the growth and invasion induced by CAFs. A,** DLD1 cells were treated with the indicated concentration of C16:0/5-FU for 48 h and assessed by annexin-V/PI assay. n=3. **B,** Animals from each test group were bioimaged (Xenogen IVIS system) by detecting the luciferase emission spectrum at 2 weeks after i.p. injection to visualize the progression of tumor growth in the peritoneum. n=4. **C,** Luciferase intensity of each test group was analyzed. Bars, mean ± SD. *, p < 0.05; **, p < 0.01; ***, p < 0.001. **D,** Proposed hypothesis of the prevention of colorectal peritoneal metastasis by palmitic acid (C16:0).

Fluoropyrimidine, or 5-fluorouracil (5-FU), is currently the most commonly used drug in the clinical treatment of CRC and forms the backbone of all first-line therapies, including both adjuvant and metastatic treatments 37. To test whether C16:0 in combination with 5-FU could increase the therapeutic effect of 5-FU *in vitro*, annexin-V/PI staining was used. The results demonstrated that C16:0 acts synergistically with 5-FU and could effectively induce cell death in CAF-CM-incubated and control CRC cells (**Fig. 9A; Fig. S9A-C**). We also used mice intraperitoneally inoculated with CAF-CM-treated HCT116 cells as an animal model to further evaluate the *in vivo* therapeutic activity of C16:0 + 5-FU in combination. As shown in **Figure 9B and C**, although C16:0 and 5-FU alone showed potent activity against CAF-CM- treated CRC cells *in vivo*, the combination of C16:0 and 5-FU significantly enhanced the therapeutic activity. Taken together, these data demonstrated that C16:0 + 5-FU is a novel drug combination that might be effective against PM-CRC.

## Discussion

Membrane fluidity can directly affect the motility and behavior of tumor cells during the different steps of metastatic dissemination 38. Alterations of the lipid composition of the plasma membrane can change the degree of plasma membrane microviscosity and fluidity 39. Zhao et al. revealed a causal association between increased membrane fluidity and metastasis potential 40. We initially focused on untargeted lipidomic profiling and found increased levels of PC with unsaturated acyl chains in clinical PM-CRC specimens. PC usually contains one or two unsaturated acyl chains, which increases membrane fluidity. Although CAFs have recently been suggested to play a tumor-suppressive role 41, 42, CAFs also play dominant roles in tumor cell growth, metastasis and chemoresistance as well as tumor immune escape 43, 44. Given the importance of the unsaturation of PC fatty acyl chains on membrane fluidity, we assumed that CAFs play a crucial role in promoting PM by increasing membrane fluidity. Then, we first discovered that the incubation of isolated CAFs with CRC cells influenced physical membrane fluidity through three independent methods: lipidomics, FRAP, and generalized polarization. Furthermore, we found that in parallel with increased membrane fluidity, CAFs promoted the invasion, metastasis and motility of CRC cells and the growth of peritoneal CRC cell implants in mice. Interestingly, increased membrane fluidity is dependent on the number of CAFs *in vitro*. Consistent with this result, high CAF abundance was linked with PM-CRC. Moreover, these findings were also supported by studies showing that the abundance of CAFs is related to prognosis in different human cancers 45. These results suggested that CAFs modify membrane fluidity via alterations in membrane lipids and that uS-FA composition may play a significant role in causing PM.

Increased cell membrane fluidity plays an important role in glucose uptake, thus increasing glycolysis. Increased glycolytic activity was found to be associated with aggressiveness and poor prognosis in various types of tumors. Although a recent study by Reinfeld et al. revealed that in the tumor microenvironment, CRC cells seem much more “addicted” to glutamine than myeloid cells and T cells, which have the greatest capacity to take up intratumoral glucose 46, our data indeed showed that CRC cells treated with CAF-CM exhibited a significant increase in glycolytic activity. Consistently, our previous study demonstrated that PM-CRC relies mostly on glycolysis 16. This pattern suggests that glycolysis drives the PM of specific tumor types. Moreover, palmitic acid (C16:0) worked synergistically with the glycolysis inhibitor 3-BrPA to significantly impair CRC cell survival after CAF-CM incubation by modulating membrane fluidity and inhibiting glucose metabolism.

Our results are partly consistent with the observation that breast CAFs induced epithelial-to-mesenchymal transition and increased cell membrane fluidity and migration in breast cancer cells through the upregulation of SCD 47. However, our data showed the opposite result: SCD expression was decreased after CAF-CM incubation. In addition, analysis of public databases revealed that lower SCD expression plays a major role in poor outcomes in CRC patients. Although the growth rate of the CRC^low SCD^ cells was slightly decreased *in vitro*, knockdown of SCD expression did not have any noticeable effect on tumor growth *in vivo*. Importantly, the minor decrease in the growth rate of the CRC^low SCD^ cells was completely reversed after CAF-CM supplementation. Importantly, the migration and invasion of CRC^low SCD^ cells were also increased in the CAF-CM-treated group. All these findings suggested a new mechanism that requires further in-depth study.

The source of exogenous MUFAs in the culture conditions was FBS supplementation. Indeed, Roongta et al. found that compared with cells grown under reduced-serum conditions (2% FBS), cells grown under normal growth conditions (10% FBS) had lower SCD protein levels 48. These data suggest that exogenous MUFA uptake plays a major role in compensating for low SCD expression. The tumor environment is a complicated system. We wondered whether CAFs excreted more FAs/lipids to fuel the proliferation and metastasis of CRC^low SCD^ cells. Untargeted metabolomics confirmed our speculation that exocrine lipids and lipid-like molecules in the CAF supernatant are taken up by CRC cells through increased membrane fluidity. Furthermore, analysis of activated CAF by incubation with [U-^13^C] stearic acid also strengthened the hypothesis that CAF-derived lipids (M+1) and exogenous lipids from the local microenvironment or de novo synthesis of endogenous lipid molecules (M+0) could be available by CRC cells.

Consistently, after CAF-CM incubation, CRC^low SCD^ cells had stronger invasion and metastatic abilities, especially cells expressing moderate amounts of SCD, indicating that exogenous lipids and SCD have a synergistic effect. A possible reason for low SCD expression in the patients with poor prognosis may be related to the biosynthesis of MUFAs by SCD; a direct approach in which exogenous lipids are obtained from the environment may be more efficiently exploited. OA (C18:1) is the most abundant uS-FA in plasma 49; therefore, we assumed that exogenous lipids or uS-FAs could compensate for low SCD expression in tumor cells, thus masking the effects of SCD deletion. Indeed, Ducheix et al. showed that dietary supplementation with OA reduced intestinal inflammation and tumor development in iSCD-/- mice 50. In addition, Vivas Garcia et al. revealed that in melanoma, SCD expression could be controlled by microphthalmia-associated transcription factor (MITF), and low SCD activity was found to impose and stabilize a dedifferentiated, invasive phenotype in MITF^Low^ cells. The effect of SCD inhibition on melanoma cells was largely reversed by the addition of the C18:1 or C16:1 MUFA to complement the lack of SCD activity 51. Notably, our previous study showed that CAFs from PM-CRC undergo lipidomic reprogramming 16. Membrane fluidity was no different in CRC cells incubated with PM or nPM CAF-CM. This may be because the number of CAFs, which excreted FAs/lipids in the cell culture supernatant, was too low to affect membrane fluidity. In support of this view, membrane fluidity significantly increased in 2-double doses of CAF-CM cultures. These results can provide some support for the ability of CAFs to promote tumor development and metastasis through lipid metabolites. All these results suggest that CAF-derived and exogenous lipids can compensate for low SCD expression and then alter lipid composition, further influencing cell membrane fluidity, which plays a profound role in promoting tumor growth and metastasis.

A variety of factors have been implicated in C16:0-mediated cellular toxicity, including ROS, ceramides, ER stress, and small nucleolar RNAs (snoRNAs) 33. Lipidomics analysis and Oil red staining showed that CAFs increased the TG intensity and promoted the accumulation of cytosolic lipid droplets in CRC cells. As a high-energy storage lipid, TG was recently identified in another study to be significantly associated with postoperative disease-free survival and lymphovascular invasion 52. Because CRC cells treated with CAF-CM exhibited large increases in the relative amounts of glycerolipids, it was logical to use C16:0 to induce toxicity and promote CRC cell apoptosis. Indeed, we demonstrated that relatively high levels of saturated glycerolipids accumulated to toxic levels and triggered the UPR. Our results also confirmed former data showing that inhibition of SCD worsened palmitate toxicity 33. Furthermore, we have revealed that inhibiting membrane fluidity and increasing lipotoxicity by C16:0 effectively suppressed colon cancer growth *in vivo*, especially when combined with 5-FU.

## Conclusion

Our study shows that colorectal CAFs increase CRC cell migration and intraperitoneal dissemination by increasing cell membrane fluidity through upregulation of the unsaturated acyl chain in PC in the cells. Furthermore, using untargeted metabolomics, we have revealed that CRC cells could take up lipids/lipid-like metabolites derived from CAFs to compensate for low SCD expression. Our findings also indicated that, on the one hand, C16:0 can modulate membrane fluidity, limit glucose metabolism, and reduce CAF-induced cell proliferation and invasiveness, while on the other hand, processes that increase FA saturation induce saturated glycerolipid-mediated lipotoxicity in cells to suppress tumor growth and intraperitoneal dissemination (**Fig. 9D**). In addition, C16:0 effectively enhanced the antitumor activity of 5-FU *in vitro* and *in vivo*. Our findings suggest that supplementation with the S-FA C16:0 may be a promising candidate as an adjuvant therapy for PM-CRC.

## Supplementary Material

Supplementary figures.

Supplementary tables.

## Abbreviations

CRC: colorectal cancer
PM-CRC: peritoneal metastasis from colorectal cancer
CRS: cytoreductive surgery
HIPEC: Hyperthermic intra-operative IntraPeritoneal Chemotherapy
CAFs: cancer-associated fibroblasts
α-SMA: α-Smooth muscle actin
uS-FAs: unsaturated fatty acids
S-FAs: saturated fatty acids
PC: phosphatidylcholine
ChE: cholesterol
PS: phosphatidylserine
PE: phosphatidylethanolamine
PI: phosphatidylinositol
SM: sphingolipids
2-NBDG: 2-Deoxy-2-[(7-nitro-2,1,3-benzoxadiazol-4-yl)amino]-D-glucose
C1-BODIPY® 500/510 C12 (BODIPY 500/510): 4,4-difluoro-5-methyl-4-bora-3a,4a-diaza-s-indacene-3-dodecanoic acid
PBS: phosphate-buffered saline
SCD: Stearoyl-CoA Desaturase 1
3-BrPA: 3-bromopyruvate
CM: conditioned medium
ECAR: extracellular acidification rate
OPLS-DA: orthogonal partial least squares discriminate analysis
5-FU: 5-fluorouracil
