# Supplementary material for: Metabolomics reveals that CAF-derived lipids promote colorectal cancer peritoneal metastasis by enhancing membrane fluidity
Source: Int J Biol Sci. 2022 Feb 21;18(5):1912–32. doi: 10.7150/ijbs.68484 (PMC8935219; doi:10.7150/ijbs.68484)
Supplement: Supplementary file 1 — Supplementary figures. [file ijbsv18p1912s1.pdf]

Supplementary figures and figure legends

Figure S1

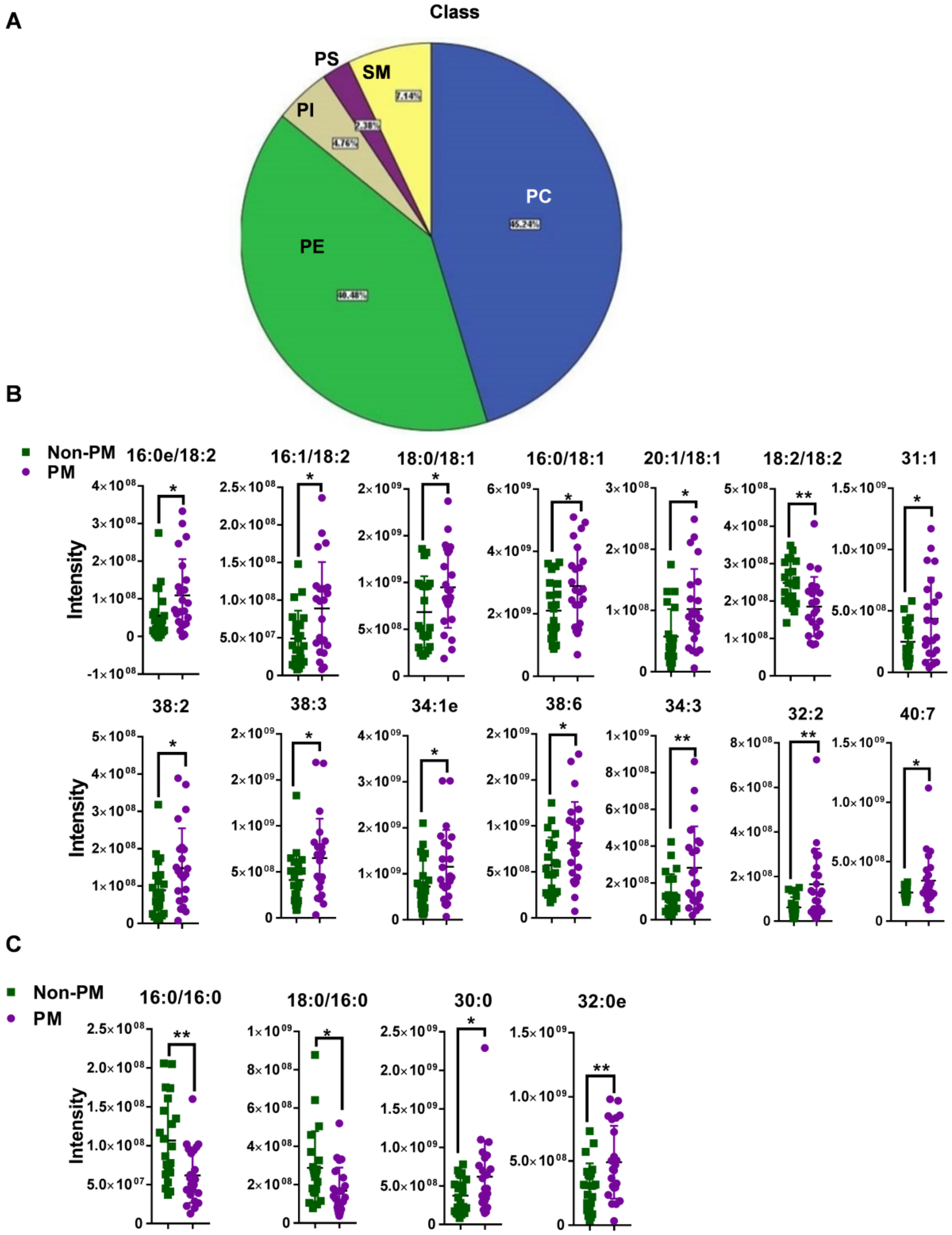

Figure S1. Altered lipids in clinical CRC specimens detected by lipidomics. A, Pie chart of lipid classes whose abundance was significantly changed. **B-C,** Intensity of phosphatidylcholine (PC) with unsaturated acyl chains (**B**) and saturated acyl chains (**C**) from clinical CRC specimens. PM, n=23; nPM, n=22. $p < 0.05$; **, $p < 0.01$.

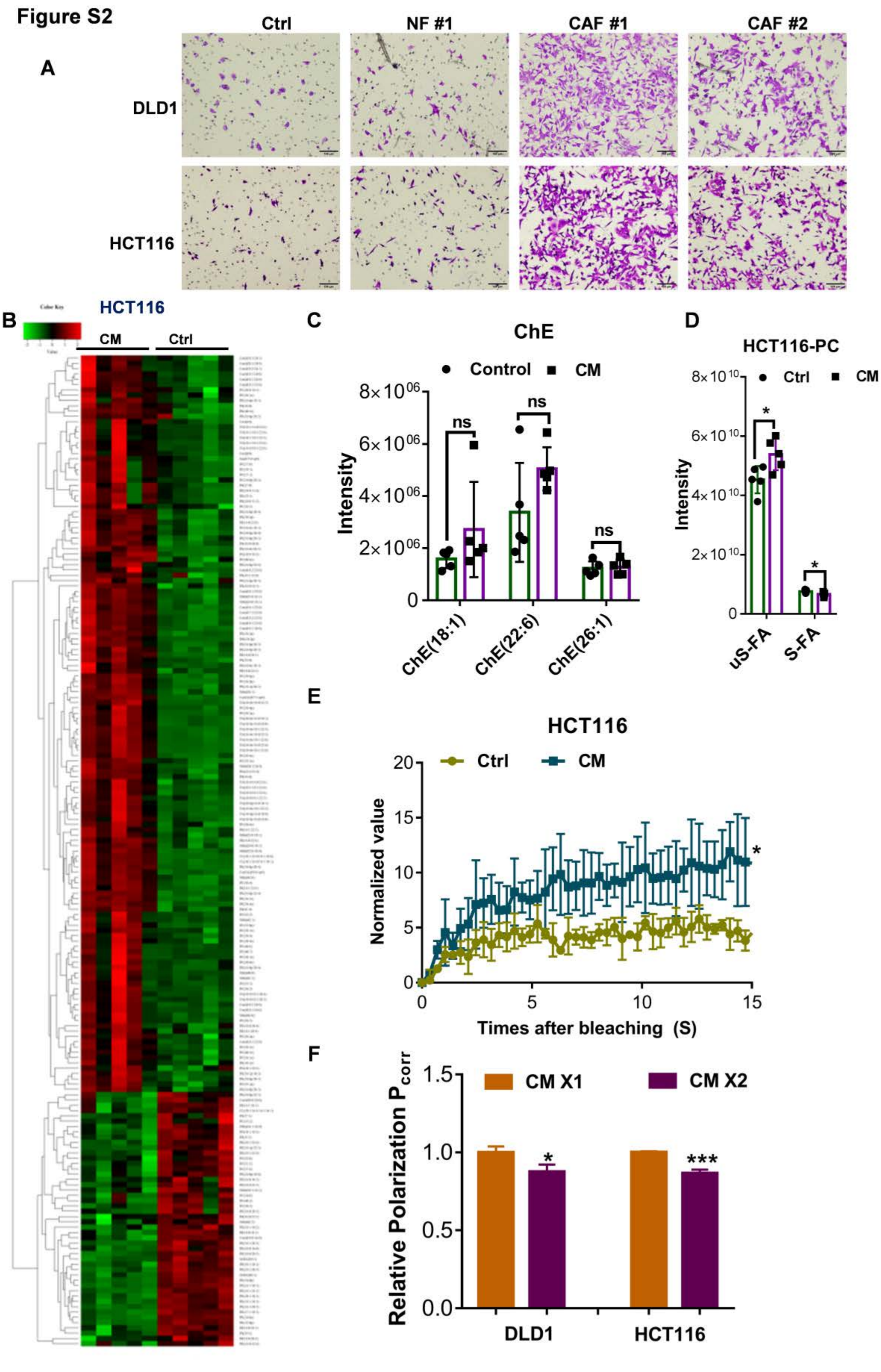

Figure S2. CAFs increased phosphatidylcholine (PC) with acyl chain unsaturation and membrane fluidity in CRC cells. **A,** Crystal violet staining to quantify the Transwell invasion of the indicated cells after 24 h of exposure to CAF-CM or NF-CM. NF, Normal fibroblast. **B,** Heat map of altered lipids in

CAF-CM-treated HCT116 cells detected by lipidomics. CAF-CM as detected by IncuCyte ZOOM. **C,** ChE (steroid) distribution in CAF-CM-incubated HCT116 cells. n=5. **D,** Intensity of PC containing saturated acyl chains and unsaturated acyl chains in CAF-CM-incubated HCT116 cells. n=5. **E,** Normalized fluorescence recovery curve of HCT116 cells cultured with control/conditioned medium. n = 3. **F**, Fluorescence polarization (Relative $Polarization_{corr}$) in CRC cells incubated with 25% CAF-CM or a double dose of CAF-CM (50%). n = 2 independent experiments with 2 CAF-CM samples from different CAF cell lines. Bars, mean ± SD. *, p < 0.05, ***, p < 0.001.

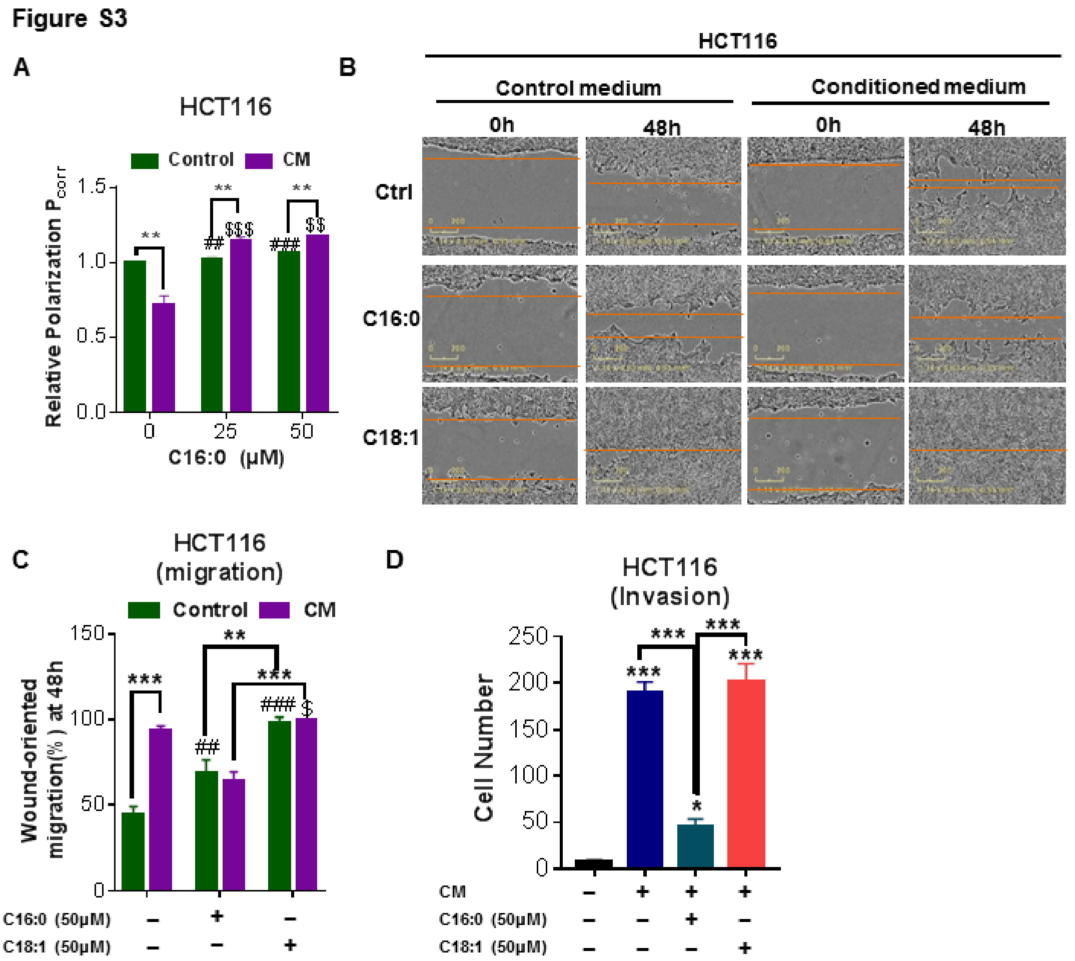

Figure S3. C16:0 inhibits CRC cell migration and invasion by decreasing membrane fluidity. A, Fluorescence polarization (Relative $Polarization_{corr}$) in CAF-CM/C16:0-treated HCT116 cells. **B,** Wound healing assay of HCT116 cells incubated with CAF-CM and treated with the indicated compounds as detected by IncuCyte ZOOM. n=3. **C,** Quantification of the wounding rates of HCT116 cells in **(B)**. # and $ indicate comparisons with the corresponding

control groups. Wound width was normalized to the initial width when the wound was created. n=3. **D,** The number of crystal violet-stained cells among HCT116 cells that invaded the Transwell. n=3. Bars, mean ± SD. n=3 *,$ $p < 0.05$; **, ##, $$ $p < 0.01$; ***, ###, $$$ $p < 0.001$.

Figure S4

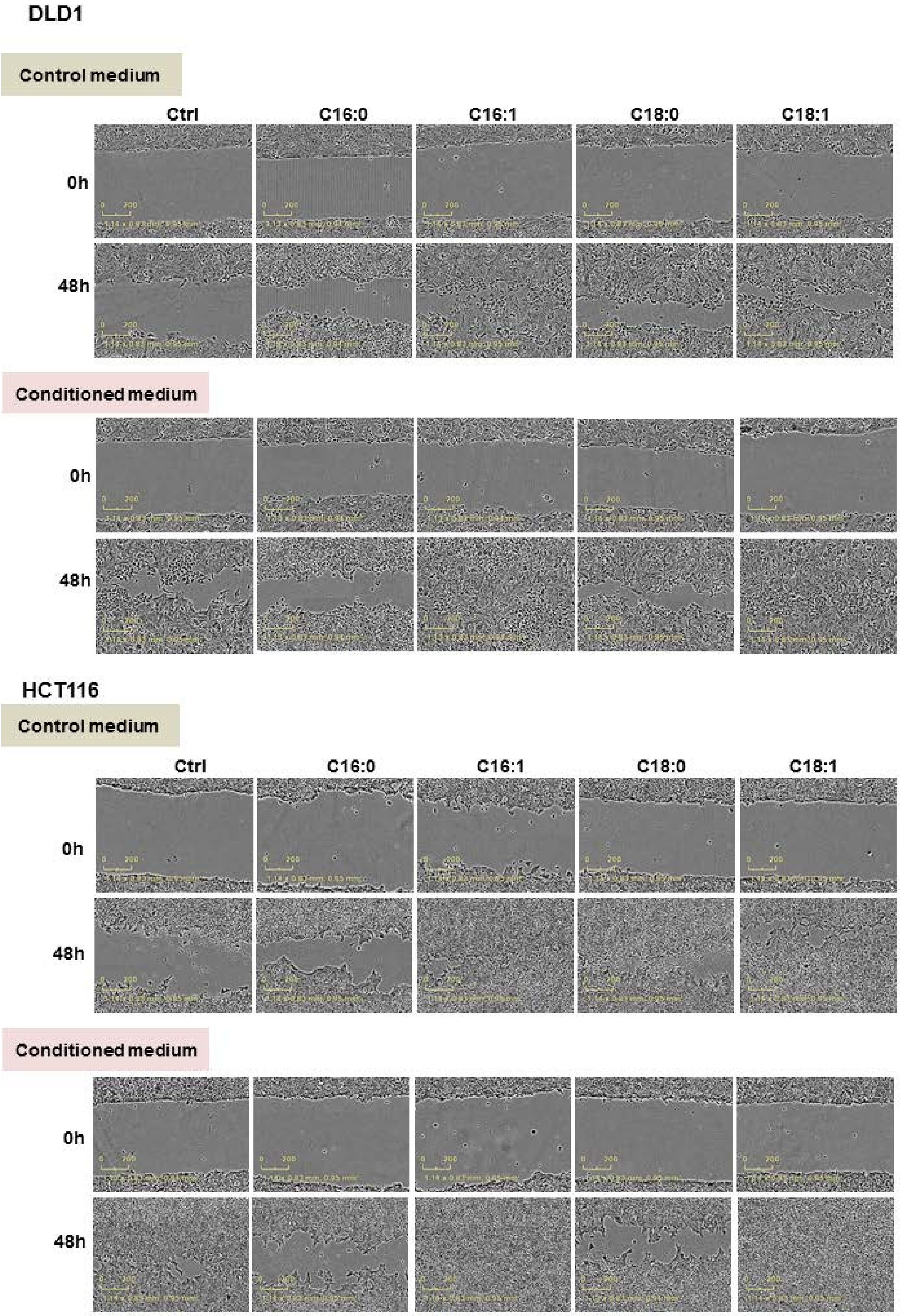

Figure S4. S-FAs inhibit CRC cell migration. Wound healing assay of DLD1/HCT116 cells incubated with CAF-CM and treated with the indicated compounds as detected by IncuCyte ZOOM. n = 2 independent experiments

with 2 CAF-CM samples from different CAF cell lines.

Figure S5

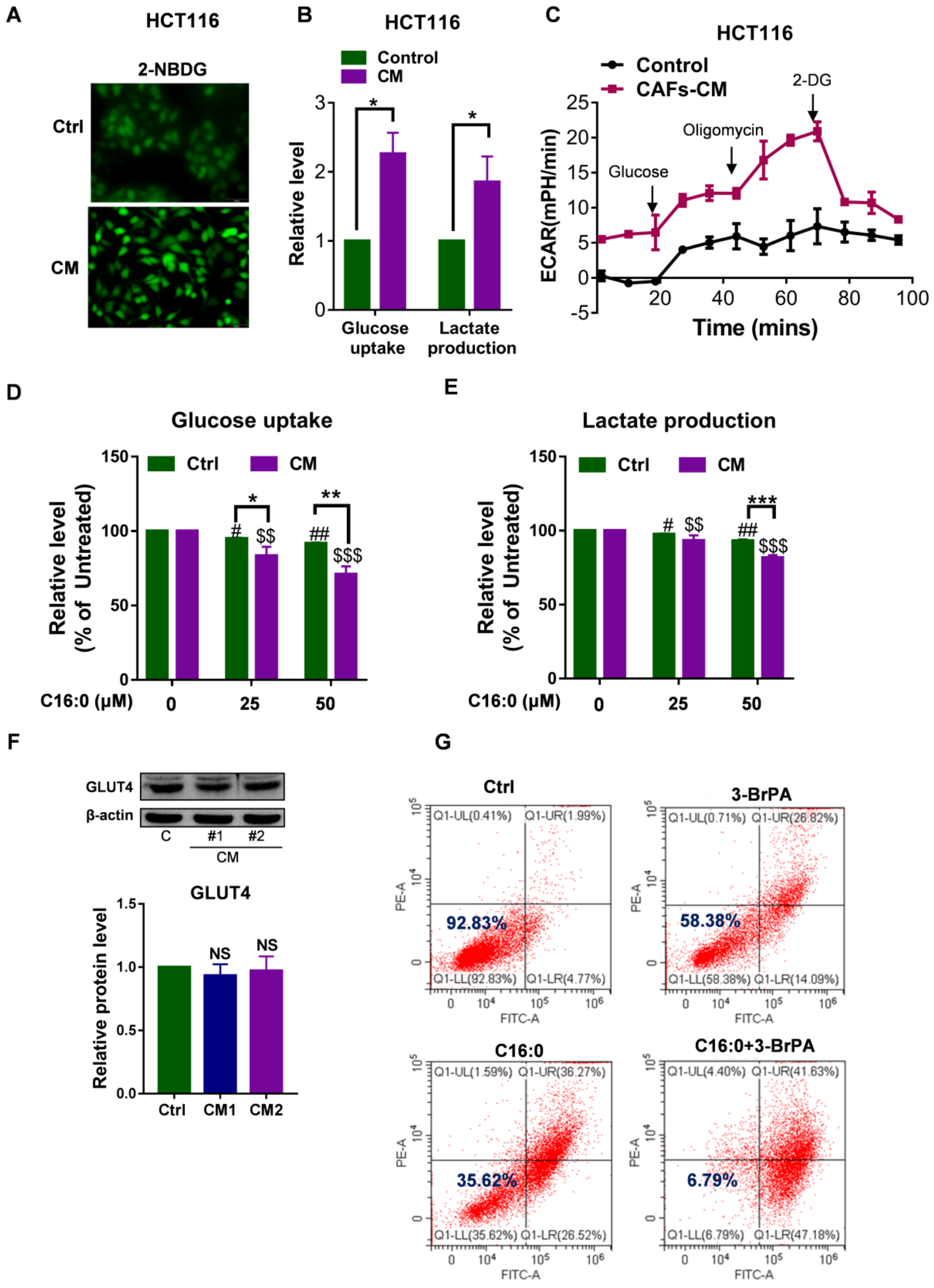

Figure S5. Increased cell membrane fluidity enhances glucose uptake and metabolism. A, Glucose uptake by HCT116 cells incubated with CAF-

CM or control medium, as visualized by 2-NBDG fluorescence. n=3. **B,** Relative levels (% of control) of glucose uptake and lactate production in HCT116 cells after incubation with CAF-CM. n=3. **C,** Impact of CAF-CM incubation on the extracellular acidification rate (ECAR). The ECAR of HCT116 cells was monitored using a Seahorse XF24 analyzer. n = 2 independent experiments with similar results. **D-E,** Glucose uptake (D) and lactate production (E) in CAF-CM-treated HCT116 cells cultured in the absence or presence of C16:0 (concentration from 0 to 50 μM). n=3. **F,** The protein level of GLUT4 in CAF-CM-treated DLD1 cells was assessed by Western blotting. n = 3. **G,** The viability of HCT116 cells incubated with CAF-CM was assessed by annexin-V/PI assay after 48 h incubation with 3-BrPA. The value in each panel indicates the % of surviving cells. n = 2 independent experiments with similar results. Bars, mean ± SD. *, # p < 0.05; **, ##, $$ p < 0.01; ***, $$$ p < 0.001.

Figure S6

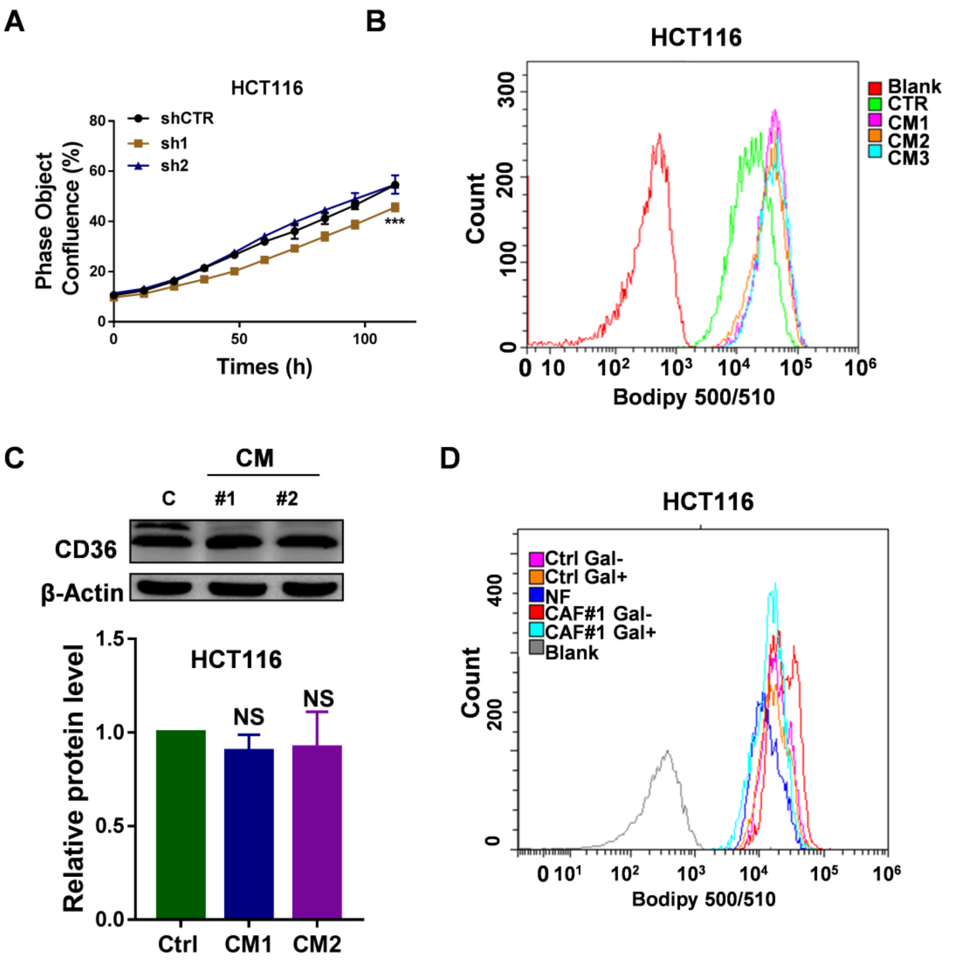

Figure S6. CAF-derived lipids boosted CRC growth and metastasis.

A, The proliferation rate of HCT116$^{low\text{-}SCD}$ cells, as evaluated by phase object confluence (%) with IncuCyte ZOOM. n=3. **B,** HCT116 cells treated as indicated were incubated with BODIPY 500 (lipid uptake). The cells were analyzed by flow cytometry. **C,** Western blot showing CD36 expression in HCT116 cells incubated with CAF-CM. **D,** The HCT116 cells treated with indicated CM were incubated with BODIPY 500/510 to analyze lipid uptake by flow cytometry. Bars, mean ± SD. ***p < 0.001; NS, not statistically significant.

Figure S7

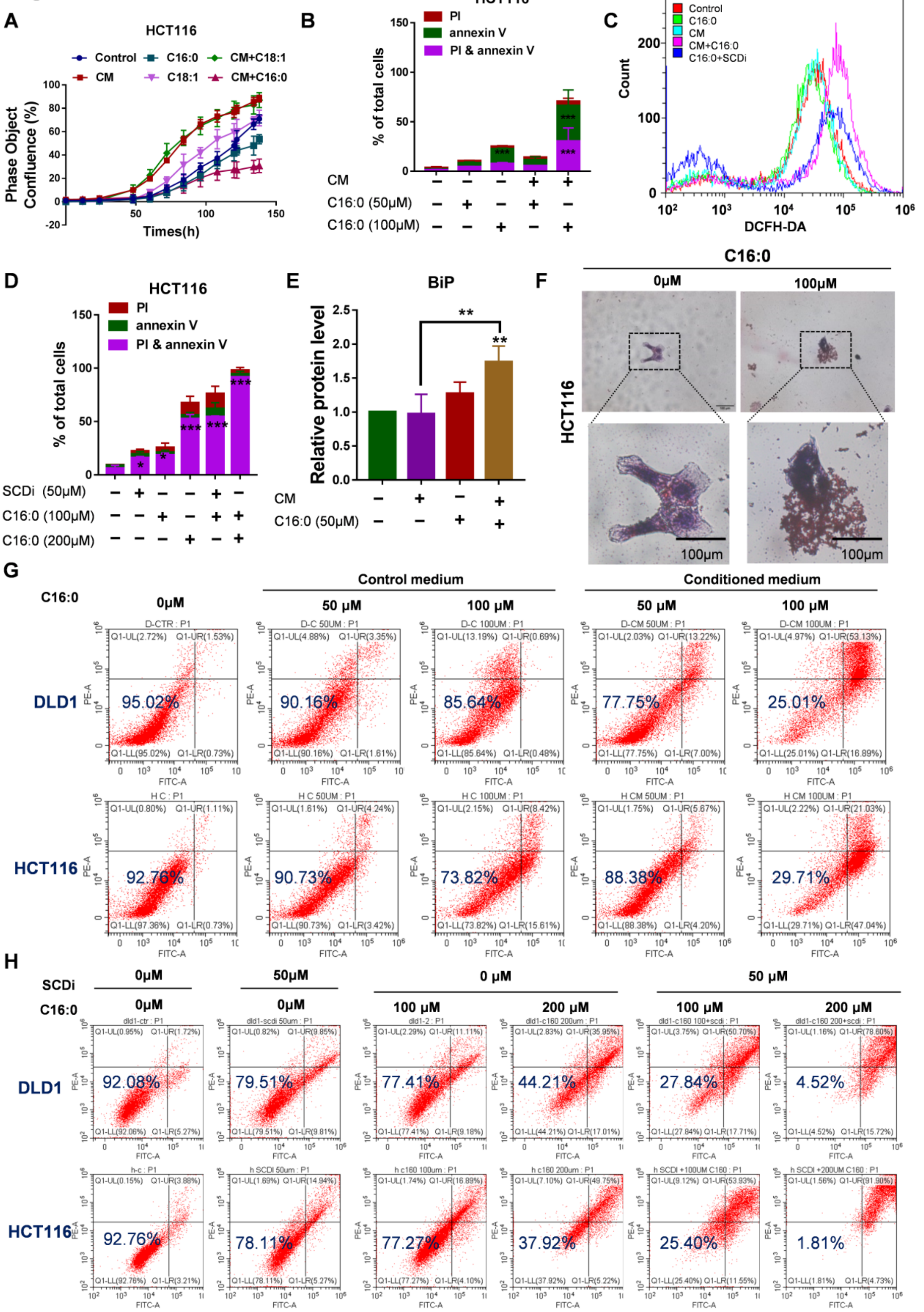

Figure S7. Treatment of cells with C16:0 increased lipotoxicity-induced CRC cell apoptosis. A, Growth curve of HCT116 cells treated with the indicated compounds. IncuCyte ZOOM software was used to process the image data for phase object confluence. n=3. **B,** C16:0 induces apoptotic cell

death in HCT116 cells. Cell viability assay of HCT116 cells treated with different concentrations of C16:0 (0, 50 and 100 μM) for 48 h. Apoptotic cells were identified by propidium iodide (PI) and annexin V staining. n=3. **C,** Reactive oxygen species (ROS) levels in HCT116 cells with or without CAF-CM incubation and SCD inhibitor treatment were detected by flow cytometry. n = 2 independent experiments with similar results. **D,** SCD inhibitor treatment increased HCT116 cell sensitivity to C16:0. Cell viability was assessed by annexin-V/PI assay. n=3. **E，** Relative protein level of BIP (GRP78 BiP) in HCT116 cells incubated with CAF-CM or after C16:0 treatment (50 μM, ~6 h) determined by Western blotting. Anti-BIP antibody (1:1000 dilution; Abcam, Cambridge, MA, USA), n=3. **F,** HCT116 cells incubated with CAF-CM and then treated with C16:0 were detected by oil red/hematoxylin staining. The cells were imaged using an inverted microscope. n = 3. **G-H,** FACS was used to detect the apoptosis of the indicated cells. Bars, mean ± SD. *, $p < 0.05$; **, $p < 0.01$; ***, $p < 0.001$.

Figure S8

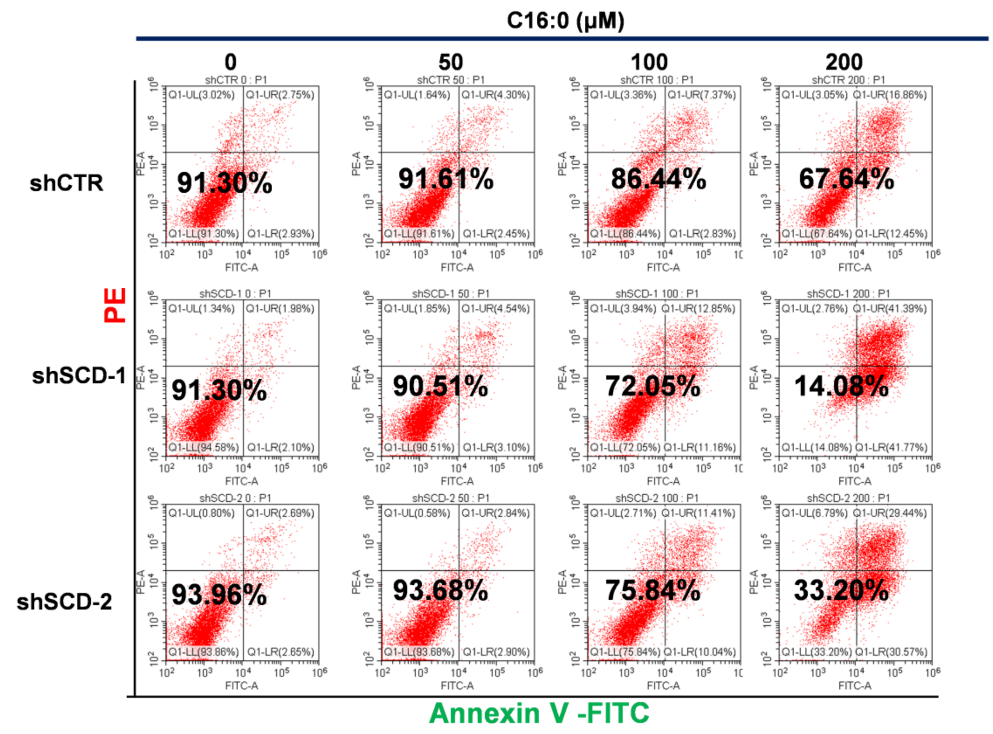

Figure S8. High doses of palmitate aggravated the lipotoxicity of CRC^{low} ^{SCD} cells. Cell viability assay of the indicated HCT116 cells treated with

different concentrations of C16:0 (0, 50, 100 and 200 μM) for 48 h and then detected by the annexin-V/PI assay. Apoptotic cells were identified by propidium iodide (PI) and annexin V staining. n=3

Figure S9

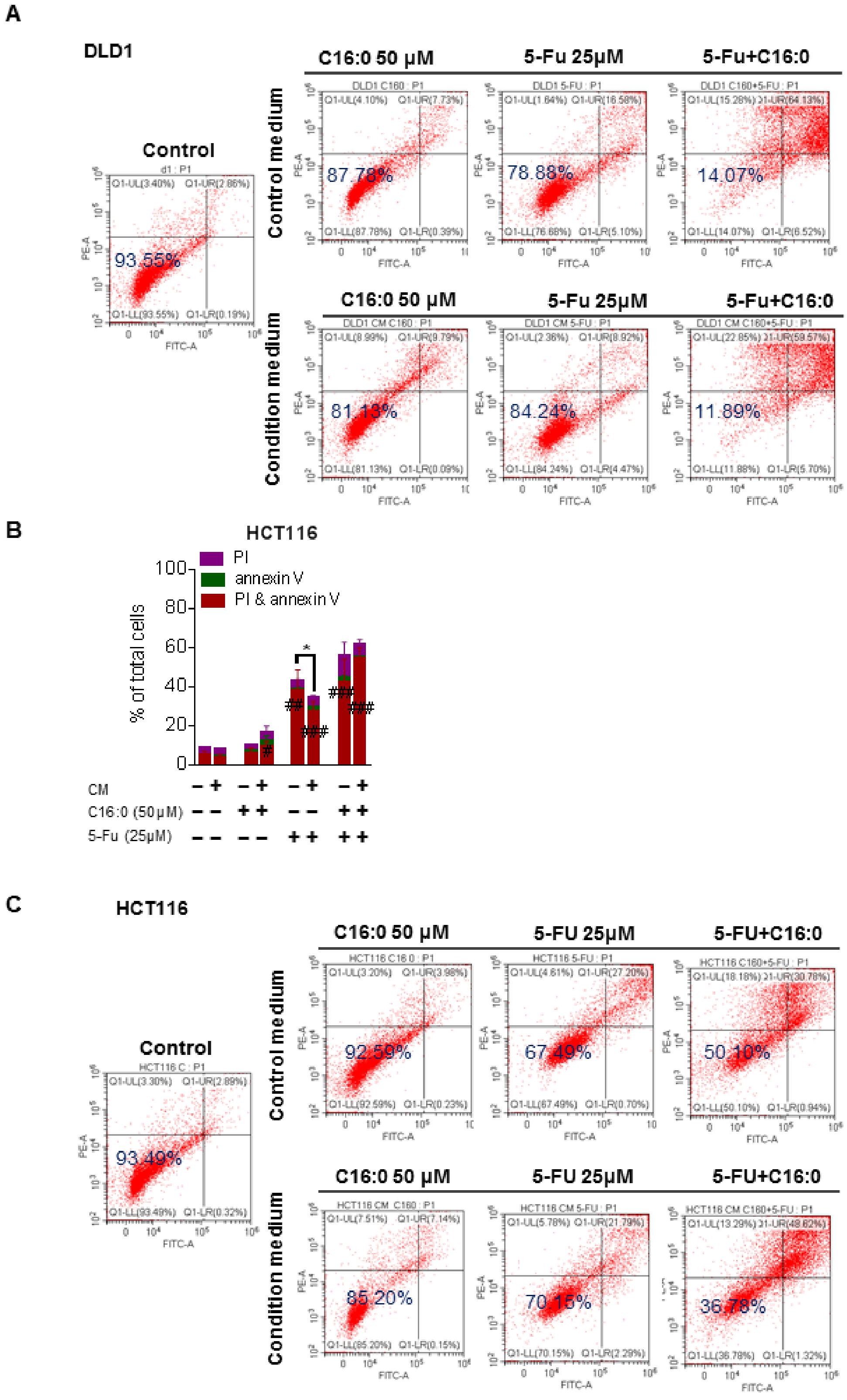

Figure S9. C16:0 acts synergistically with 5-FU to effectively kill CRC cells incubated with CAF-CM *in vitro*. A, FACS was used to detect the apoptosis of the indicated cells. DLD1 cells were treated with the indicated

concentration of C16:0/5-FU for 48 h and subjected to the annexin-V/PI assay. **B,** C16:0/5-FU induced apoptotic cell death in HCT116 cells. n=3. **C,** FACS was used to detect the apoptosis of the indicated cells. HCT116 cells were treated with the indicated concentration of C16:0/5-FU for 48 h and subjected to the annexin-V/PI assay. Bars, mean ± SD. *, $p < 0.05$; **, $p < 0.01$; ***, $p < 0.001$.
